# Phosphate‐Templated Encapsulation of a {Co^II^_4_O_4_} Cubane in Germanotungstates as Carbon‐Free Homogeneous Water Oxidation Photocatalysts

**DOI:** 10.1002/cssc.202100506

**Published:** 2021-05-11

**Authors:** Emir Al‐Sayed, Sreejith P. Nandan, Elias Tanuhadi, Gerald Giester, Marco Arrigoni, Georg K. H. Madsen, Alexey Cherevan, Dominik Eder, Annette Rompel

**Affiliations:** ^1^ Fakultät für Chemie Institut für Biophysikalische Chemie Universität Wien Althanstraße 14 1090 Wien Austria; ^2^ Institute of Materials Chemistry TU Wien Getreidemarkt 9 Vienna 1060 Austria; ^3^ Fakultät für Geowissenschaften Geographie und Astronomie Institut für Mineralogie und Kristallographie Universität Wien Althanstraße 14 1090 Wien Austria

**Keywords:** cobalt, homogeneous catalysis, nanoclusters, photochemistry, sustainable energy

## Abstract

The ever‐growing interest in sustainable energy sources leads to a search for an efficient, stable, and inexpensive homogeneous water oxidation catalyst (WOC). Herein, the PO_4_^3−^ templated synthesis of three abundant‐metal‐based germanotungstate (GT) clusters Na_15_[Ge_4_PCo_4_(H_2_O)_2_W_24_O_94_] ⋅ 38H_2_O (Co_4_), Na_2.5_K_17.5_[Ge_3_PCo_9_(OH)_5_(H_2_O)_4_W_30_O_115_] ⋅ 45H_2_O (Co_9_), Na_6_K_16_[Ge_4_P_4_Co_20_(OH)_14_(H_2_O)_18_W_36_O_150_] ⋅ 61H_2_O (Co_20_) with non‐, quasi‐, or full cubane motifs structurally strongly reminiscent of the naturally occurring {Mn_4_Ca} oxygen evolving complex (OEC) in photosystem II was achieved. Under the conditions tested, all three GT‐scaffolds were active molecular WOCs, with Co_9_ and Co_20_ outperforming the well‐known Na_10_[Co_4_(H_2_O)_2_(PW_9_O_34_)_2_] {Co_4_P_2_W_18_} by a factor of 2 as shown by a direct comparison of their turnover numbers (TONs). With TONs up to 159.9 and a turnover frequency of 0.608 s^−1^ Co_9_ currently represents the fastest Co‐GT‐based WOC, and photoluminescence emission spectroscopy provided insights into its photocatalytic WOC mechanism. Cyclic voltammetry, dynamic light scattering, UV/Vis and IR spectroscopy showed recyclability and integrity of the catalysts under the applied conditions. The experimental results were supported by computational studies, which highlighted that the facilitated oxidation of Co_9_ was due to the higher energy of its highest occupied molecular orbital electrons as compared to Co_4_.

## Graphical abstract

**Water oxidation**: The stabilization of non‐, quasi‐ and full‐cubane topologies in three Co^II^ germanotungstates (Co‐GT) [Ge_4_PCo_4_(H_2_O)_2_W_24_O_94_]^15−^ (Co_4_), [Ge_3_PCo_9_(OH)_5_(H_2_O)_4_W_30_O_115_]^20−^ (Co_9_), [Ge_4_P_4_Co_20_(OH)_14_(H_2_O)_18_W_36_O_150_]^22−^ (Co_20_) via PO_4_^3−^ and GT lacunary ligands is reported. The Co‐GTs are active molecular water oxidation catalysts with Co_9_ and Co_20_ outperforming the non‐cubane [Co_4_(H_2_O)_2_(PW_9_O_34_)_2_]^10−^ by a factor of 2, showing the structural and functional similarity of the GT‐encapsulated cubanes to the {Mn_4_Ca} oxygen evolving complex.

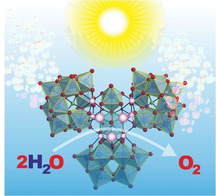

## Introduction

The development of an artificial, efficient, stable, and inexpensive homogeneous water oxidation catalyst (WOC), which can mimic the natural photosynthesis process to meet mankind's growing energy demands, is of utmost interest.^[1]^ Over the past decade, 3d‐ and 4d‐doped polyoxotungstates (POTs) have been reported as promising all‐inorganic water oxidation catalysts (POT‐WOCs), which, contrary to their organic counterparts, can withstand the harsh oxidizing conditions of the water oxidation half‐reaction.^[2]^ Starting in 2008, Bonchio and co‐workers^[3]^ and Hill and co‐workers^[4]^ independently reported the ruthenium‐containing POT [{Ru_4_O_4_(OH)_2_(H_2_O)_4_}(γ‐SiW_10_O_36_)_2_]^10−^ (Ru_4_) as the first efficient POT for visible‐light‐driven homogeneous water oxidation catalysis [turnover frequency (TOF)=0.25 s^−1^]. In Ru_4_, the WOC active metal core consists of a tetrahedron of four ruthenium centers sandwiched between two dilacunary [γ‐SiW_10_O_36_]^8−[5]^ POT units, thereby resembling the core {Mn_4_Ca} in the oxygen‐evolving complex (OEC) in photosystem II (Figure S6).^[6]^ In 2010, the activity of the cobalt‐containing Weakley dimer [Co_4_(H_2_O)_2_(PW_9_O_34_)_2_]^10−^ as the first non‐precious metal‐based homogeneous POT‐WOC was reported by Hill and co‐workers.^[7]^ Under visible‐light irradiation and in the presence of [Ru(bpy)_3_]^2+^ as a photosensitizer and S_2_O_8_^2−^ as an oxidant, WOC activity with turnover numbers (TONs) up to 220 and TOFs up to 5 s^−1^ was observed. Since then, attention was given to Co‐containing polyoxometalates (POMs) in terms of their stability and activity as WOCs under various reaction conditions with special interest in cobalt‐cubane (Table S2) scaffolds as bio‐inspired cost‐effective WOCs with enhanced photocatalytic activity.[^8^, ^9^] Significant progress was achieved by Wei et al., who reported the octa‐cobalt‐substituted silicotungstate [Co_8_(OH)_6_(H_2_O)_2_(CO_3_)_3_(*A*‐α‐SiW_9_O_34_)_2_]^16−^ as the currently fastest Co‐substituted bio‐inspired POT‐WOC (TOF=10 s^−1^, Table S3).^[10]^ At the same time, Wang and co‐workers investigated the photocatalytic WOC performance of a series of isostructural cubane‐incorporating hexadecanuclear Co‐POTs [{Co_4_(OH)_3_PO_4_}_4_(XW_9_O_34_)_4_]^*n*−^ (X=P^V^, As^V^; *n*=28; X=Si^IV^, Ge^IV^; *n*=32) showing that the type of primary heteroatom in the lacunary POM unit efficiently modulates their overall redox properties and WOC activity with Ge^IV^ (TOF=0.105 s^−1^) and As^V^ (TOF=0.053 s^−1^) containing representatives exhibiting the highest WOC activity.^[11]^ Taken together the structural and compositional features of the most promising POT‐WOCs recently reported in literature and considering the low number of two cobalt cubane germanotungstates with proven WOC activity reported so far (Table S2), we chose the structurally simple yet versatile PO_4_^3−^ group for the synthesis of all‐inorganic cobalt germanotungstate (GT) clusters as OEC models. Herein, we report the PO_4_^3−^‐templated stabilization of a series of all‐inorganic Co‐GT, Na_15_[Ge_4_PCo_4_(H_2_O)_2_W_24_O_94_] ⋅ 38H_2_O (Co_4_), Na_2.5_K_17.5_[Ge_3_PCo_9_(OH)_5_(H_2_O)_4_W_30_O_115_] ⋅ 45H_2_O (Co_9_) and Na_6_K_16_[Ge_4_P_4_Co_20_(OH)_14_(H_2_O)_18_W_36_O_150_] ⋅ 61H_2_O (Co_20_) with non‐, quasi‐, or full {Co^II^_4_O_4_} cubane motifs and their activity in visible‐light‐driven water oxidation. With 20 incorporated Co^II^ metal centers Co_20_ comprises the highest reported number of incorporated metal centers in a cubane‐encapsulating POT (Table S2). Under visible‐light irradiation and in the presence of [Ru(bpy)_3_]^2+^ as a photosensitizer and S_2_O_8_^2−^ as an oxidant, WOC activity with TONs up to 159.9 and TOFs up to 0.608 s^−1^ for Co_9_ in borate buffer at pH 8.0 was detected, which to the best of our knowledge represents the currently fastest Co‐GT‐based homogeneous POT‐WOC (Table S3).

## Results and Discussion

### Synthesis and structure

**Figure 1:**
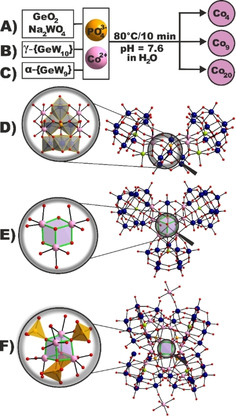
Schematic representation of the building block dependent synthesis of (A) Co_4_, (B) Co_9_, and (C) Co_20_, which are templated by PO_4_^3−^ after heat activation. Ball‐and‐stick representation of (D) Co_4_, (E) Co_9_ enclosing the quasi‐ {Co^II^_3_O_4_} cubane, and (F) Co_20_ incorporating the PO_4_^3−^‐stabilized {Co^II^_4_O_4_} cubane. Color code: {WO_6_}, dark blue; Co^II^, pink; Ge^IV^, lime; P^V^, light orange; O, red, {W_3_O_13_} triads of Co_4_, grey octahedra.

Co_4_, Co_9_ and Co_20_ are synthesized in a non‐buffered aqueous solution by adjusting the pH of a reaction mixture containing the corresponding tungsten source (GeO_2_ and Na_2_WO_4_ for Co_4_, K_8_[γ‐GeW_10_O_36_] ⋅ 6H_2_O^[12]^ for Co_9_ and K_8_Na_2_[*A‐*α*‐*GeW_9_O_34_] ⋅ 25H_2_O^[13]^ for Co_20_) and CoCl_2_ to pH 7.6 via addition of Na_3_PO_4_. Heat activation of the PO_4_^3−^ group enabling its coordination to the Co‐oxo cores and subsequent filtration of the cooled reaction mixture results in single crystals of Co_4_, Co_9_ and Co_20_ at 20 °C (CCDC 1876468‐1876470, Figure 1A–F). Single‐crystal X‐ray diffraction (SXRD) studies revealed that Co_4_ and Co_20_ crystallize in the monoclinic space groups *P*2_1_/*c* and *C*2/*c*, whereas Co_9_ crystallizes in the triclinic space group *P* $\bar{1}$ (Tables S5–S10). In all three compounds at least one PO_4_^3−^ group stabilizes the POT scaffold. For Co_4_ a PO_4_^3−^ group connects two trigonal edge‐shared {W_3_O_13_} fragments to the four octahedrally coordinated Co^II^ centers, which are encapsulated by two *B*‐α‐{GeW_9_} units and two germanium octahedrons located externally in the structure (Figure 1D, inset). Note, Co_4_ is the first reported structure with germanium octahedrons in a pure inorganic GT. In Co_9_, the PO_4_^3−^ group connects two α‐{Co_2_GeW_10_} Keggin moieties with one exchangeable Co^II^‐coordinated aqua ligand per α‐{Co_2_GeW_10_} unit and one virtual “γ‐{Co_3_GeW_9_}” building block (Figure S7) to a single {WO_6_} octahedron. Two octahedrally coordinated Co^II^O_5_(H_2_O) centers encapsulated by the PO_4_^3−^ and the single {WO_6_} unit complete the trimeric polyanion by forming a {Co^II^_3_O_4_(H_2_O)_2_} quasi‐cubane (Figure 1E, inset) featuring two exchangeable aqua ligands, thereby resulting in a total number of four Co^II^ positions exhibiting exchangeable aqua ligands, suitable for water molecules to supposedly coordinate and subsequently get oxidized. The architecture of Co_20_ presents a tetrameric aggregate of four α‐{Co_3_GeW_9_} units linking to a central {Co^II^_4_O_4_} cubane, which is geometrically closely related to the {Mn_3_CaO_4_} cubane of the OEC in PSII^[6b]^ (Figure S6) and stabilized by four PO_4_^3−^ groups (Figure 1F, inset). Four covalently bound Co^II^ octahedra located in the POT‐encapsulated Co‐oxo core and externally as antenna‐like metal centers^[14]^ complete the Co_20_ framework. The compounds’ elemental composition and homogeneity was determined by elemental analysis, IR spectroscopy (Figure S2), thermogravimetric analysis (TGA; Figures S3–S5, Table S4), and powder XRD (PXRD; Figures S8–S10). To probe the solution stability of Co_4_, Co_9_ and Co_20_, time‐dependent UV/Vis spectra were recorded at various pH conditions. It should be mentioned that UV/Vis experiments on Co_4_, Co_9_ and Co_20_ in the absorption range for octahedrally coordinated Co^II^ centers could not be performed due to the low solubility of the Co‐GTs and the strong domination of the p_π_(O_t_)→d_π*_(W) ligand‐to‐metal charge‐transfer (LMCT) transitions, which is a problem commonly encountered in POM chemistry.^[15]^ Therefore, the precatalytic POT stability was assessed by investigating the LMCT transitions in the tungsten absorption range. The UV/Vis spectra of Co_4_, Co_9_ and Co_20_ display an absorption maximum at approximately 205 nm with a shoulder at approximately 250 nm corresponding to the Keggin‐type framework in aqueous solution (Figures S17–S19).^[16]^

**Figure 2:**
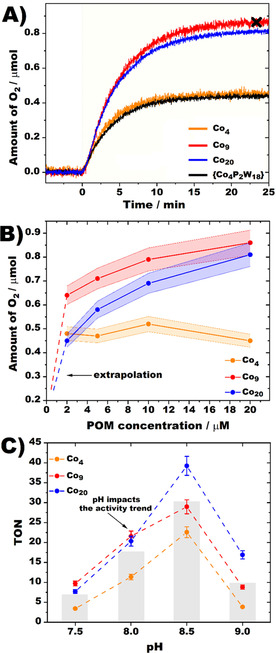
(A) As‐recorded O_2_ evolution profiles for Co_4_, Co_9_ and Co_20_ compounds along with the reference {Co_4_P_2_W_18_}, measured from 20 μm POM solutions buffered in 80 mm borate buffer at pH 8 and containing Na_2_S_2_O_8_ (5 mm) and [Ru(bpy)_3_]^2+^ (1 mm) as an oxidant and a photosensitizer, respectively. (B) Recorded oxygen evolution amounts as a function of POM catalyst concentrations measured for 2, 5, 10 and 20 μm values. A summary of as‐recorded profiles is shown in Figure S23. (C) Amount of generated O_2_ plotted as TONs as a function of pH (7.5–9). The bars indicate an average O_2_ amount generated by all three catalysts at a certain pH to demonstrate a cumulative effect of pH on activity. For all conditions, a monochromatic visible LED (*λ*_max_=445±13 nm) was chosen as light source to trigger photocatalytic reaction.

Acidification of an unbuffered solution of Co_20_ (pH=0.7) led to stepwise degradation of the POT framework shown by the disappearance of the shoulder at approximately 250 nm (Figure S20), whereas all UV/Vis spectra remain unchanged in 80 mm sodium borate buffer (pH=7.5–9) solutions for at least 2 h mimicking the photocatalytic conditions (Figures S17–S19), which suggests pre‐catalytic stability of Co_4_, Co_9_ and Co_20_ until O_2_ saturation is reached in the WOC experiments (Figure 2A).

Cyclic voltametric measurements were performed on Co_4_, Co_9_ and Co_20_ (vs. Ag/AgCl, in 80 mm sodium borate buffer pH=8) thereby revealing Co^2+^/Co^3+^ oxidation waves in the range E≈0.73–0.89 V^[17]^ (Figure S21) occurring at lower potentials than those for other Co‐POM‐WOC systems at pH≥8 (>0.9 V),[^11^, ^12^, ^13^, ^14^, ^15^, ^16^, ^17^, ^18^] whereas the Co(NO_3_)_2_ reference solution gives the most intense peaks for Co^2+^/Co^3+^ oxidation (at 0.84 V) as well as for water oxidation (at 1.37 V) (Figure S22) showing that the cobalt centers incorporated in the germanotungstates do not behave like free cobalt ions (Table S12) and additionally indicating their pre‐catalytic stability.

### Visible light‐driven water oxidation

To probe the photocatalytic WOC activity of Co_4_, Co_9_ and Co_20_, 80 mm aqueous borate buffer solutions (pH=7.5–9) of POTs (concentrations ranging from 2 to 20 μm) containing 1 mm Ru[bpy]_3_Cl_2_ (bpy=2,2’‐bipyridine) as the photosensitizer (PS) and 5 mm Na_2_S_2_O_8_ as a sacrificial agent (SA) were used. Note that various parameters such as the shape of the reaction vessel, light intensity, stirring rate as well as the ratio of gaseous head space to total volume render a direct comparison of the WOC performance of POTs tested in different catalytic systems difficult.^[19c]^ Hence, the literature‐known Weakley‐type [Co_4_(H_2_O)_2_(PW_9_O_34_)_2_]^10−^ {Co_4_P_2_W_18_} was used as a benchmark POT‐WOC.[^7^, ^11^, ^19^] Figure 2A shows as‐recorded O_2_ evolution profiles of 20 μm Co_4_, Co_9_, Co_20_ and {Co_4_P_2_W_18_} solutions at pH 8 and indicates WOC activity for all compounds, with Co_9_ and Co_20_ reaching TONs of 21.6 and 20.4, thus outperforming {Co_4_P_2_W_18_} (TON=10.9 under elsewise identical conditions) by a factor of 2 (Table 1). Initial TOF values of 0.047 (Co_4_), 0.069 (Co_9_), and 0.068 s^−1^ (Co_20_), respectively, are determined from the derivative plots, which are comparable to those of other reported Co‐POM‐WOCs like [{Co_4_(OH)_3_(PO_4_)}_4_(SiW_9_O_34_)_4_]^32−^ (TOF=0.053 s^−1^) and [{Co_4_(OH)_3_(PO_4_)}_4_(GeW_9_O_34_)_4_]^32−^ (TOF=0.105 s^−1^), both exhibiting structurally similar attributes like an incorporated {Co^II^_4_O_4_} cubane motif (Table S3).^[11]^ Figure 2B demonstrates the amount of generated O_2_ as a function of POM concentration and reveals a direct activity correlation with increasing concentration for Co_9_ and Co_20_ (red and blue trends), in contrast to Co_4_ (orange trend), which indicates that factors other than the catalyst concentration, such as the amount of sacrificial agent or photosensitizer, control its WOC performance (Figures S23, S24). At pH=8 Co_9_ is the most active WOC species with a rather small advantage over Co_20_, which gets more pronounced at lower catalyst concentrations (Table 1) with Co_9_ reaching TONs up to 159 and a TOF=0.608 s^−1^, currently representing the highest TOF for a Co‐GT‐based homogeneous POM‐WOC. The higher WOC activity of Co_9_ compared to both Co_4_ and Co_20_ may have its origin in the enhanced accessibility of the Co^II^ centers, which leads to supposedly facilitated coordination and oxidation of water molecules on the catalytic centers (Figures 1E, S7, Table S11).^[10]^ pH‐dependent WOC studies (pH=7.5–9) revealed higher O_2_ evolution amounts and TOF values with increasing pH values from 7.5 to 8.5 (Figure 2C), which is related to thermodynamic aspects of water oxidation catalysis according to the Nernst equation,^[20]^ and partial POM deprotonation that results in its enhanced interaction with the photosensitizer.^[21]^ A strong activity drop at pH=9 (indicated with grey bars in Figure 2C) is related to [Ru(bpy)_3_]^2+^ degradation.^[2a]^ Overall, the highest WOC activity was determined at pH=8.5, indicating a trade‐off between activity and PS‐stability at these conditions. At pH=8.5 (in contrast to the data obtained at pH=8) Co_20_ (Table 1) greatly outperforms Co_9_. According to bond valence sum (BVS) studies, Co_20_ is the most protonated species in the solid state of the three (H_25_Co_20_, H_13_Co_9_, H_4_Co_4_, Table S11), which in turn means that the effect of its deprotonation at higher pH values is more pronounced leading to a stronger WOC increase. This activity trend implies that the ultimate performance of Co_4_, Co_9_ and Co_20_ is affected by the number of Co^II^ centers, their accessibility, and the extent of POM‐PS pairing, and can be governed by each of these factors depending on the pH.

**Table 1:** Summary table showing average TON and initial TOF values for Co_4_, Co_9_, Co_20_ and the Weakley‐type POM {Co_4_P_2_W_18_} as well as the O_2_ yield generated by the respective POM with varying concentration (2–20 μm) in borate buffer [80 mm], pH=8.0.

Co‐POM (μm)	O_2_ [μmol] (TON)	TOF [s^−1^]	O_2_ yield [%]
Co_4_ (2)	0.480 (120.50)	0.422	9.60
Co_9_ (2)	0.640 (159.90)	0.608	12.80
Co_20_ (2)	0.450 (111.40)	0.405	4.50
Co_4_ (5)	0.470 (46.90)	0.148	9.40
Co_9_ (5)	0.710 (71.30)	0.263	14.20
Co_20_ (5)	0.575 (57.50)	0.189	28.40
Co_4_ (10)	0.520 (25.90)	0.091	10.40
Co_9_ (10)	0.790 (39.30)	0.125	15.80
Co_20_ (10)	0.690 (34.40)	0.105	13.80
Co_4_ (20)	0.450 (11.30)	0.047	9.00
Co_9_ (20)	0.860 (21.60)	0.069	17.20
Co_20_ (20)	0.810 (20.40)	0.068	16.20
{Co_4_P_2_W_18_} (20)	0.430 (10.90)	0.015	8.60

### Post‐catalytic stability studies

The stability of molecular WOCs is a topic of current interest due to the possibility of the WOCs to decompose into catalytically active oxide nanoparticles.^[22]^ Note that ^183^W or ^31^P NMR measurements on Co_4_, Co_9_ and Co_20_ could not be performed, due to the low solubility and strong paramagnetic nature of the incorporated Co^II^ metal centers.^[23]^ Hence, various reloading experiments and post‐catalytic characterizations were conducted to demonstrate that O_2_ evolution is indeed triggered by the investigated Co‐GT and to verify their integrity under turnover conditions.^[24]^

First, blank WOC experiments in pure water and those performed in the absence of PS and SA showed no activity (Figure S25A), indicating the validity of the experimental setup. Only a small activity was recorded in the absence of any Co‐GT (≈20 % of the activity of Co_9_ at pH=8) (Figure S25A), which suggests a negligible contribution of direct water oxidation by the PS* as a side reaction and is in line with previous reports.^[25]^ Moreover, a reference WOC experiment using the unsubstituted [PW_12_O_40_]^3−^ Keggin^[26]^ POT instead of Co_4_, Co_9_ or Co_20_ yielded a similarly low activity (Figure S25B) thereby excluding in‐situ formed tungsten‐based species to be responsible for the observed WOC activity. Second, after O_2_ level reached saturation (point x in Figure 2A), the reaction solution was re‐loaded with PS and SA. Figure S26 containing the summary WOC data demonstrates that this second illumination cycle triggers additional O_2_ evolution, which suggests that the observed WOC saturation is not a result of POM deactivation or degradation but can rather be related to the depletion of the other WOC‐solution components such as the [Ru(bpy)_3_]^2+^ or the S_2_O_8_^2−^, and additionally indicates recyclability of the POM‐WOCs. Third, following an established procedure,^[24c]^ a toluene solution of tetra‐*n*‐heptylammonium nitrate (THpANO_3_) was used to quantitatively extract Co_4_, Co_9_ and Co_20_ from the respective post‐catalytic solutions (see Figure S27 along with discussion). As this selective procedure does not extract CoO_*x*_ or Co^2+^_aq_, we analyzed the remaining aqueous phases with X‐ray fluorescence spectroscopy (XRF) to elucidate the potential leaching of Co into the reaction mixture under photocatalytic conditions. The XRF spectra of the solutions look similar and did not show any Co traces (Figure S27, Table S13), which indicates that the Co‐GT underwent neither decomposition (e. g., into Co^2+^) nor degradation (e. g., into CoO_*x*_), processes that have been previously identified to be responsible for WOC performance of other POMs and under different experimental conditions.^[22]^ Fourth, dynamic light scattering (DLS) was performed on 20 μm solutions of Co_4_, Co_9_ or Co_20_, [Ru(bpy)_3_]^2+^ (1 mm) and S_2_O_8_^2‐^ (5 mm) in 80 mm borate buffer (pH=8) after 30 min irradiation. The DLS measurements showed no nanoparticles after photocatalytic water oxidation (Figure S28). In addition, the same experiments were conducted using 20 μm Co(NO_3_)_2_ ⋅ 6H_2_O instead of the corresponding Co‐GT and here nanoparticles with a diameter of approximately 26.4 nm were detected (Figure S28). This is in line with the XRF experiments (Figure S27, Table S13) and confirms that, in contrast to the Co(NO_3_)_2_ ⋅ 6H_2_O system, no metal hydroxide/oxide nanoparticles (especially cobalt hydroxide/oxide nanoparticles) are generated via hydrolytic decomposition of Co_4_, Co_9_ and Co_20_, nor through detachment of the four covalently bound Co^II^ antenna ligands present in Co_20_ after the photocatalytic experiments. Finally, attenuated total reflectance (ATR)‐IR spectra of Co_4_, Co_9_ and Co_20_ were recorded after the photocatalytic experiments and subsequent precipitation with cesium chloride. These clearly show the characteristic W−O−W bridging and terminal W=O vibrations in the tungsten fingerprint area from 300–1000 cm^−1^ (Figures S29–S31), which indicates the solution of the polyanions is stable under turnover conditions and represents an established method frequently used for the post‐catalytic study of POMs.[^23^, ^27^]

### Mechanistic studies

Photoluminescence (PL) emission spectroscopy was employed to investigate the photocatalytic WOC mechanism and to understand the electron transfer kinetics between the reaction solution components. Figure S32 reveals that the PL emission of [Ru(bpy)_3_]^2+^ is quenched by both Na_2_S_2_O_8_ and Co_9_ in a linear Stern‐Volmer behavior depending on their concentrations (Figure S33). The calculated rate constant for the oxidative quenching by Na_2_S_2_O_8_ is 45 times lower than that of the reductive quenching by Co_9_. Considering (a) the much higher Na_2_S_2_O_8_ concentration (5 mm) as compared to Co_9_ (2–20 μm) present in the WOC reaction solution, and (b) the use of the borate buffer that weakens the formation of an ion pair between polyanionic Co_9_ and cationic [Ru(bpy)_3_]^2+^,^[17]^ oxidative quenching dominates under the photocatalytic conditions. This conclusion is strongly confirmed by time‐resolved PL decay profiles. Figure S34 indicates that in the presence of 10 mm Na_2_S_2_O_8_ and 20 μm of Co_9_, the original PS* lifetime of approximately 395 ns decreases to approximately 264 and 339 ns, respectively, illustrating that both quenchers speed up the decay kinetics of *Ru(bpy)_3_^2+^ and that S_2_O_8_^2−^ takes up electrons more efficiently. Thus, it can be suggested that during the photocatalytic process, Co_9_ is oxidized by the oxidized form of the PS (Scheme S1), which is in accordance with previously reported POM‐WOCs.[^17^, ^28^]

### Computational studies

**Figure 3:**
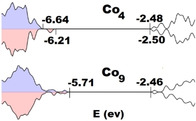
Density of states for the *α* (blue) and *β* (red) electrons in the Co_4_ and Co_9_. Shaded areas represent occupied states.

**Figure 4:**
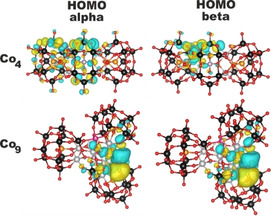
Representation of the *α* and *β* HOMO for Co_4_ and Co_9_. Color code: W^VI^, black; Co^II^, pink; Ge^IV^, orange; P^V^, light purple; O, red; Zn^II^, silver.

The electronic structures of Co_4_ and Co_9_ were analyzed by means of density functional theory (DFT) simulations.^[29]^ All cobalt atoms in Co_4_ and Co_9_ were considered in the +2 oxidation state with a formal d^7^ high‐spin configuration. Both polyanions exhibit a complex electronic structure with 12 (Co_4_) and 27 (Co_9_) unpaired electrons, respectively. Therefore, the corresponding models were simplified by replacing all except one of the Co^II^ ions with Zn^II^ resulting in a single catalytic site with a formal spin state characterized by three unpaired electrons. It has been recently shown that such an approximation does not affect the eigenvalues of the frontier molecular orbitals considerably, thereby representing a good trade‐off between the computational costs involved for the study of such systems and a reasonable level of accuracy.^[30]^ The Co active site in the high spin state forms a distorted octahedron with the neighboring O atoms. This local environment is very similar in Co_4_ and Co_9_ and the calculated results are in good agreement with the values measured by XRD. In Co_4_, the calculated values of the Co−O bond lengths present in the octahedron are around 2.10±0.08 Å (range 2.00–2.19 Å), in good agreement with the value of 2.10±0.05 Å (range 2.00–2.20 Å) observed in the crystal structures. In Co_9_, these bonds have a comparable length of approximately 2.12±0.05 Å (range 2.06–2.20 Å), compared with the experimental value of 2.11±0.06 Å (range 1.96–2.27 Å). Considering the electronic configuration of Co_4_ and Co_9_, Figure 3 shows the calculated density of states for the alpha and beta electrons obtained from the calculated molecular orbitals eigenvalues after applying a Gaussian smearing. The highest occupied molecular orbitals (HOMO) of Co_9_ lie higher in energy than those of the Co_4_ system, while the lowest unoccupied molecular orbitals (LUMO) have comparable energies. In particular, the calculated eigenvalues for the HOMO electrons in Co_4_ have values of −6.64 and −6.21 eV for the *α* and *β* electrons, respectively, while for Co_9_ both alpha and beta HOMO electrons have an energy of around −5.71 eV (Figures 3, 4). Regarding the LUMO orbitals, they are almost degenerate for both systems: for Co_4_ the *α* and *β* energies have values of −2.48 and −2.50 eV, respectively, while for Co_9_ both *α* and *β* LUMO electrons have an energy of −2.46 eV. Consequently, the HOMO‐LUMO gap for the Co_4_ system is 4.16 eV for the *α* electrons and 3.71 eV for the *β* ones. For Co_9_, the HOMO‐LUMO gap is around 3.25 eV for both spin orientations. The observed HOMO‐LUMO gap trends are additionally supported by experimental estimations done by performing diffuse reflectance spectroscopy (Figures S11–S14) and cyclic voltammetry measurements (Figures S15, S16) on Co_4_ and Co_9_.

Our mechanistic photoluminescence findings suggested oxidative quenching to be the dominant WOC pathway, which implies that Co_9_ undergoes oxidation by [Ru(bpy)_3_]^3+^ on the third stage of the catalytic cycle (Scheme S1). According to DFT calculations, the higher energy of the HOMO electrons in Co_9_ indicates that it is thermodynamically more prone to oxidation as compared to Co_4_. Considering the higher TOF values obtained for the Co_9_, these complementary data suggest that POT oxidation may be one of the rate‐limiting processes and that a careful adjustment of the POM/PS redox properties can be used as a tool to optimize the ultimate WOC performance.

## Conclusion

The phosphate templated stabilization of non‐ (Co_4_), {Co^II^_3_O_4_} (Co_9_) quasi‐ and {Co^II^_4_O_4_} (Co_20_) full cubane structural motifs in germanotungstates as cost‐effective, active, and stable molecular water oxidation catalysts (WOCs) mimicking the naturally occurring oxygen‐evolving complex (OEC) is highlighted. The functional properties of the PO_4_^3−^‐stabilized cubane motifs are shown by the doubled WOC activity of Co_9_ and Co_20_ compared to the Weakley‐type (non‐cubane) benchmark {Co_4_P_2_W_18_}. Comprehensive mechanistic and electronic structure studies by photoluminescence and density functional theory suggest an oxidative quenching WOC mechanism and relate the ultimate performance of Co_9_ to its redox levels. The confirmed stability and recyclability of Co_9_ and Co_20_ encourage their immobilization in matrices leading to heterogeneous photocatalytic materials as a subject of future applications.

## Experimental Section

### Preparation of Na_15_[Ge_4_PCo_4_(H_2_O)_2_W_24_O_94_] ⋅ 38H_2_O (Co_4_)

Na_2_WO_4_ ⋅ 2H_2_O (1.82 g, 5.52 mmol) and GeO_2_ (0.054 g, 0.52 mmol) were dissolved in 40 mL water and subsequently CoCl_2_ ⋅ 6H_2_O (0.073 g, 0.31 mmol) was added. The pH was quickly adjusted from approximately 8.5 to 7.4 by the addition of 1 m HCl (≈5 mL) and then further to approximately 4.8 by 37 % HCl. The pH of the clear red solution was then raised to approximately 7.9 by adding solid Na_3_PO_4_ (≈0.28 g, 1.708 mmol) and adjusted to approximately 7.6 with 1 m HCl. The reaction mixture was then heated for approximately 10 min at 80 °C and filtered. The dark red solution was stored in an open beaker at room temperature. After about 2–3 weeks, red rhombohedral‐shaped crystals formed. After recrystallizing in water for several times some X‐ray‐suitable crystals were obtained. Yield: 0.015 g (1 % based on W). Anal. Calcd. [%] for Co_4_: Na, 4.62; Co, 3.89; P, 0.41; W, 59.09; Ge, 3.81; Found: Na, 4.70; Co, 3.80; P, 0.15; W, 53.1; Ge, 2.15. Selected Fourier‐transform (FT)IR bands: $\tilde{\nu }$ =3340 (br), 1617 (m), 1088 (w), 1038 (w), 934 (m), 865 (s), 820 (m), 759 (s), 650 (s), 583 (s), 509 (s), 437 cm^−1^ (s).

### Preparation of Na_2.5_K_17.5_[Ge_3_PCo_9_(OH)_5_(H_2_O)_4_W_30_O_115_] ⋅ 45H_2_O (Co_9_)

K_8_[γ‐GeW_10_O_36_] ⋅ 6H_2_O^[12]^ (0.5 g, 0,172 mmol) was dissolved in 15 mL H_2_O and subsequently CoCl_2_ ⋅ 6H_2_O (0.119 g, 0.5 mmol) was added. The pH of the clear dark red solution was then adjusted to 7.7 by adding solid Na_3_PO_4_ (≈0.07 g, 0.427 mmol). A color change to magenta occurred and slight precipitate formed. The reaction mixture was then heated for approximately 10 min at 80 °C, filtered and stored partially closed in a temperature‐controlled crystallization room (19±1 °C). After about one month, dark red‐violet block‐shaped for X‐ray‐suitable crystals formed in addition to very lightly colored crystalline precipitate. Yield: 0.14 g (25 % based on W). Anal. Calcd. [%] for Co_9_: Na, 0.71; K, 6.85; Co, 5.46; P, 0.32; W, 56.8; Ge, 2.24; Found: Na, 0.94; K, 6.42; Co, 4.15; P, 0.53; W, 49.5; Ge, 2.00. Selected FTIR bands: $\tilde{\nu }$ =3364 (s), 3217 (s), 1620 (m), 1096 (w), 1033 (w), 1004 (w), 935 (m), 860 (m), 811 (m), 748 (m), 670 (m), 509 (m), 434 cm^−1^ (m).

### Preparation of Na_6_K_16_[Ge_4_P_4_Co_20_(OH)_14_(H_2_O)_18_W_36_O_150_] ⋅ 61H_2_O (Co_20_)

K_8_Na_2_[*A‐*α*‐*GeW_9_O_34_] ⋅ 25H_2_O^[13]^ (0.5 g, 0.16 mmol) was dissolved in 40 mL H_2_O and subsequently CoCl_2_ ⋅ 6H_2_O (0.119 g, 0.47 mmol) was added. After stirring for approximately 20 min at room temperature, the pH of the clear red solution was then raised to 7.6 by adding solid Na_3_PO_4_ (≈0.07 g, 0.427 mmol). The reaction mixture was then heated for approximately 10 min at 80–85 °C, filtered and stored in an open beaker in a temperature‐controlled crystallization room (19±1 °C). After 2–3 days, red needle‐shaped crystals formed. After recrystallizing in water and slow evaporation at +4 °C X‐ray‐suitable crystals were obtained. Yield: 0.08 g (15 % based on W). Anal. Calcd. [%] for Co_20_: Na, 0.51; K, 3.79; Co, 10.11; P, 0.92; W, 49.37; Ge, 2.17; Found: Na, 1.02; K, 4.64; Co, 7.09; P, 0.88; W, 45.1; Ge, 2.06. Selected FTIR bands: $\tilde{\nu }$ =3340 (br), 1614 (w), 1088 (w), 927 (m), 863 (m), 783 (s), 650 (s), 584 (s), 518 (s), 452 (s), 438 cm^−1^ (s).

### Preparation and characterization of Na_10_[Co_4_(H_2_O)_2_(α‐PW_9_O_34_)_2_] ⋅ 27H_2_O ({Co_4_P_2_W_18_})

{Co_4_P_2_W_18_} was prepared according to the literature procedure reported by Hill and co‐workers.^[7]^ The identity of {Co_4_P_2_W_18_} was proven by single‐crystal XRD (Table S1) and electrospray ionization mass spectrometry (ESI‐MS) (Figure S1).

### Visible‐light‐driven water oxidation

A homogeneous solution of 80 mm aqueous borate buffer (pH=7.5, 8, 8.5 or 9) containing 1.0 mm of [Ru(bpy)_3_]Cl_2_, 5.0 mm of Na_2_S_2_O_8_ and POM catalyst with concentrations varying from 2–20 μm was prepared in a two‐necked closed glass reactor equipped with an outer water‐cooling jacket. This solution was then deaerated using a flow of argon and later irradiated with a monochromatic visible LED (*λ*_max_=445±13 nm) to trigger a photocatalytic reaction. The oxygen evolution was followed in situ using an optical oxygen meter (FireStingO2, Pyroscience, Germany) and a needle‐like oxygen‐sensitive optical sensor (OXF900PT‐OI) with a working principle based on the quenching of the REDFLASH indicator (immobilized on the sensor tip) luminescence caused by a collision between oxygen molecules and the indicator. In a single experiment, the oxygen sensor was inserted through a Viton septum placed in a screw cap on one of the necks of the reactor. The O_2_ concentration was measured directly in %O_2_ and was later converted to μmol and TONs based on the control experiments and the ideal gas equation. Initial TOFs were calculated as the maximum derivative (obtained from the “μmol of O_2_ vs. time” plots) divided by the number of moles of the catalyst.

### Computational details

All DFT calculations were performed employing the computer code NWChen.^[31]^ The exchange‐correlation functional was approximated by employing the Becke‐3‐parameter‐Lee–Yang–Parr functional (B3LYP).^[32]^ The core electrons of the Co, P, Ge, W, and Zn atoms were described by the LANL2DZ effective core potential^[33]^ and the corresponding basis set was used for the valence electrons. Electrons of the H atoms were described by the 6–31G basis set and the 3–21G basis set was used for the O atoms.^[34]^ Employing the larger 6–31G basis set for the O atoms affects the HOMO‐LUMO gap of Co_4_ by around the 0.2 %, while the eigenvalues corresponding to the HOMO molecular orbitals change by slightly less than the 5 %. We therefore performed all calculations employing the smaller basis set. In both Co_4_ and Co_9_, all Co atoms except one were substituted by Zn atoms. This procedure was performed to simplify the complex magnetic structure endowed by the presence of multiple Co^2+^ cations. Haider et al. have shown that for similar Co‐containing polyanions, such substitution affects the eigenvalues of the frontier molecular orbitals only marginally.^[30]^ The remaining Co atom in the polyanion was considered to be in the high‐spin quadruplet configuration. All structures were optimized in water, described with the conductor‐like screening (COSMO) continuum solvation model.^[35]^ The permittivity of water was set to 78.36 and the radii of the atomic‐centered spheres used to construct the molecule‐shaped cavity were set to the corresponding atomic Van der Waals radii. The structures of all compounds were relaxed within the solvent model to a minimum of the potential energy surface employing a quasi‐Newton optimization method.

## Conflict of interest

The authors declare no conflict of interest.

## Supporting information

As a service to our authors and readers, this journal provides supporting information supplied by the authors. Such materials are peer reviewed and may be re‐organized for online delivery, but are not copy‐edited or typeset. Technical support issues arising from supporting information (other than missing files) should be addressed to the authors.

Supplementary
