# Supplementary material for: Phosphate‐Templated Encapsulation of a {CoII 4O4} Cubane in Germanotungstates as Carbon‐Free Homogeneous Water Oxidation Photocatalysts
Source: ChemSusChem. 2021 May 11;14(12):2529–36. doi: 10.1002/cssc.202100506 (PMC8251812; doi:10.1002/cssc.202100506)
Supplement: Supplementary file 1 — Supplementary [file CSSC-14-2529-s001.pdf]

ChemSusChem

Supporting Information

Phosphate-Templated Encapsulation of a $\{Co^{II}_4O_4\}$ Cubane in Germanotungstates as Carbon-Free Homogeneous Water Oxidation Photocatalysts

Emir Al-Sayed[+], Sreejith P. Nandan[+], Elias Tanuhadi[+], Gerald Giester, Marco Arrigoni, Georg K. H. Madsen, Alexey Cherevan, Dominik Eder,* and Annette Rompel*© 2021 The Authors. ChemSusChem published by Wiley-VCH GmbH. This is an open access article under the terms of the Creative Commons Attribution License, which permits use, distribution and reproduction in any medium, provided the original work is properly cited.

Content

1. General Information 3

2. Experimental Procedure 5

3. IR-spectroscopy 9

4. Thermogravimetric Analysis 10

5. Single-Crystal X-ray Diffraction 13

6. Bond-Valence Summation (BVS) Analysis 17

7. Powder X-ray Diffraction (PXRD) 18

8. Optical Band Gap 21

8.1. Estimation of the band gap E_g using diffuse reflectance spectroscopy (DRS) 21

8.2. Estimation of band gap position using cyclic voltammetry (CV) 25

9. Pre-catalytic studies 28

10. Visible light-driven water oxidation 35

10.1. Impact of POM-WOC (**Co_4**, **Co_9** and **Co_{20}**) concentrations 35

10.2. Impact of $Na_2S_2O_8$ and $[Ru(bpy)_3]^{2+}$ concentrations 36

11. POM WOC activity and integrity 37

11.1 Co-GT recyclability 38

11.2 X-ray fluorescence analysis: Extraction procedure of **Co_4**, **Co_9** and **Co_{20}** for X-ray fluorescence analysis 39

11.3 Post-catalytic POM-precipitation for subsequent analysis with FTIR 42

12. Photoluminescence (PL) emission spectroscopy 44

13. References 47

1. General Information

All reagents were purchased commercially and used without further purification. Na_2WO_4 • $2H_2O$ (Sigma Aldrich ≥99%), GeO_2 (Sigma Aldrich ≥99.9%), $CoCl_2$ • $6H_2O$ (Sigma Aldrich reagent grade), Na_3PO_4 (Riedel de Haën ≥94%). $K_8Na_2[A\text{-}\alpha\text{-}GeW_9O_{34}]$ • $25H_2O$, $K_8[\gamma\text{-}GeW_{10}O_{36}]$ • $6H_2O$, $Na_{10}[Co_4(H_2O)_2(\alpha\text{-}PW_9O_{34})_2]$ • $27H_2O$ and $Na_3[PW_{12}O_{40}]$·• $12H_2O$ were prepared according to published procedures and characterized by IR spectroscopy.[1]

Elemental analysis: Inductively coupled plasma-mass spectrometry was performed for the determination of the elements P, Co, Ge, and W using a Perkin Elmer Elan 6000 ICP-MS in aqueous solutions containing 2 % ultrapure HNO_3. Flame atomic absorption spectroscopy was performed on a Perkin Elmer 1100 Flame AAS to determine the exact concentrations of Na and K.

Attenuated total reflection Fourier−transform Infrared Spectroscopy: All FTIR spectra were recorded on a Bruker Vertex 70 IR Spectrometer equipped with a single−reflection diamond−ATR unit. Frequencies are given in cm^{-1}, intensities denoted as w = weak, m = medium, s = strong, br = broad.

Thermogravimetric analysis (TGA): TGA was performed on a Mettler SDTA851e Thermogravimetric Analyzer under nitrogen flow with a heating rate of 5 K min^{-1} in the region 298−1023 K.

Single crystal X-ray diffraction (SXRD): The X-ray intensity data were measured on a Bruker Apex2 diffractometer equipped with a CCD area detector and an Incoatec Microfocus Source IμS (30 W, multilayer mirror, Mo-K_α) at 200K (OxfordCryosystems Cryostream 800 Plus). The structures were solved by Direct methods and refined by full-matrix least-squares techniques. Non-hydrogen atoms were refined with anisotropic displacement parameters. The following software was used: Frame integration, Bruker SAINT software package [2] using a narrow-frame algorithm, Absorption correction, SADABS [3], structure solution, SHELXL-2013 [4], refinement, SHELXL-2013 [4], OLEX2 [5] and SHELXLE [6]. Crystal data, data collection parameters and structure refinement details are given in **Tables S5 – S10**. Further crystallographic data have been deposited with the Cambridge Crystallographic Data Centre and can be obtained free of charge from the Cambridge Crystallographic Data Centre, 12 Union Road, Cambridge CB2 1EZ, UK; fax: +44 1223 336033, citing the title of this paper and the corresponding **CCDC 1876468 – 1876470**.

Powder X-ray diffraction was performed on an EMPYREAN diffractometer system using Cu Kα radiation (λ = 1.540598), a PIXcel3D-Medipix3 1 × 1 detector (used as a scanning line detector) and a divergence slit fixed at 0.1 mm. The scan range was from 5° to 50° (2θ).

Diffuse reflectance spectroscopy (DRS) was performed on a Jasco V-670 UV-Vis photo spectrometer using a diffuse reflectance unit containing an Ulbricht-sphere. The powdered samples were fixed in the micro sample holder with a diameter of 3 mm and $MgSO_4$ was used as a standard.

Mass spectrometry was performed with an ESI−Qq−oaRTOF supplied by Bruker Daltonics Ltd. Bruker Daltonics Data Analysis software was used to analyze the results. The measurement was performed in a 1:1 mixture of $H_2O/CH_3CN/MeOH$, collected in negative

ion mode and with the spectrometer calibrated with the standard tune−mix to give an accuracy of ca. 5 ppm in the region of m/z 300−3000.

UV–Vis spectroscopy: UV−vis spectra were collected on a Shimadzu UV 1800 spectrophotometer. The spectra were recorded in 80 mM sodium borate buffer (pH 7.5-9).

Cyclic voltammetry: All measurements were carried out using a HEKA PG 390 potentiostat at ambient temperature (25°C). A conventional three-electrode arrangement in a glass cell of 10 mL capacity was used. A 2 mm diameter glassy carbon disk electrode as the working electrode (GCE), a platinum wire as the counter electrode and an Ag/AgCl electrode as the reference electrode were used. All solutions were deoxygenated using argon gas prior to electrochemical experiments.

X-ray fluorescence analysis: Chemical analysis with Total-reflection X-Ray Fluorescence (TXRF) was performed to analyze the aqueous phase of reaction solution (after extracting out the POMs into organic phase using $THpANO_3$ cations) using Wobistrax X-ray fluorescence analyzer. This spectrometer operates with a total reflection geometry using an energy-dispersive Si detector, and the measurements were done with monochromatized Rh-Kα excitation mode (20.2 keV) at 50 kV and 0.7 mA, for 200 s live time. Liquid samples (5 mL) were taken and a fixed concentration (10 ppm) of Ga was added as an internal standard to help quantification of elements present. The solution was then vortexed, dropped (5 μL) onto quartz reflectors and dried on a hot plate, and the obtained film was then analyzed.

Dynamic light scattering: DLS analyses were performed on an ALV/CGS-3 compact goniometer system equipped with a He - Ne laser as a light source (@632.8 nm, power of 22 mW), at a goniometer angle of 90° and room temperature. To avoid contaminations, all samples were filtered by using 0.45 mm polypropylene filter (VWR) up to three times prior to measurement.

Photoluminescence (PL) emission spectroscopy: Photoluminescence steady state measurements of $[Ru(bpy)_3]Cl_2$ solution (with and without quenchers) were carried out using Picoquant FluoTime 300 spectrophotometer. The excitation source was ozone-free Xe arc lamp (300 W power), coupled with a double grating monochromator. The detection system includes a PMA Hybrid 07 detector with a high-resolution double monochromator. For all the steady state measurements, the excitation wavelength used was 445 nm (2.79 eV photon energy). The concentration of the $[Ru(bpy)_3]Cl_2$ solution was set to be in the range where inner filter effect does not occur. Time-resolved PL spectra were acquired using a 377 nm laser wavelength, at a detection wavelength of 620 nm for all the solutions. The PL data was collected and fitted using EasyTau2 software.

2. Experimental Procedure

Table S1. Single-crystal X-ray analysis of **{$Co_4P_2W_{18}$}** reveal the same cell-parameters as reported proving successful re-synthesis of the compound. Published cell parameters [1] are given in brackets for comparison.

**Sum formula**	**$Na_{10}[Co_4(H_2O)_2(\alpha\text{-}PW_9O_{34})_2] \cdot 27H_2O$**
**a [Å]**	11.65 [11.539(3)]
**b [Å]**	12.95 [12.807(4)]
**c [Å]**	17.45 [17.259(5)]
**α [°]**	98.98 [98.019(4)]
**β [°]**	107.92 [106.639(4)]
**γ [°]**	112.54 [111.205(4)]
**V [Å³]**	2201 [2192.30]
**Space group**	**P-1**

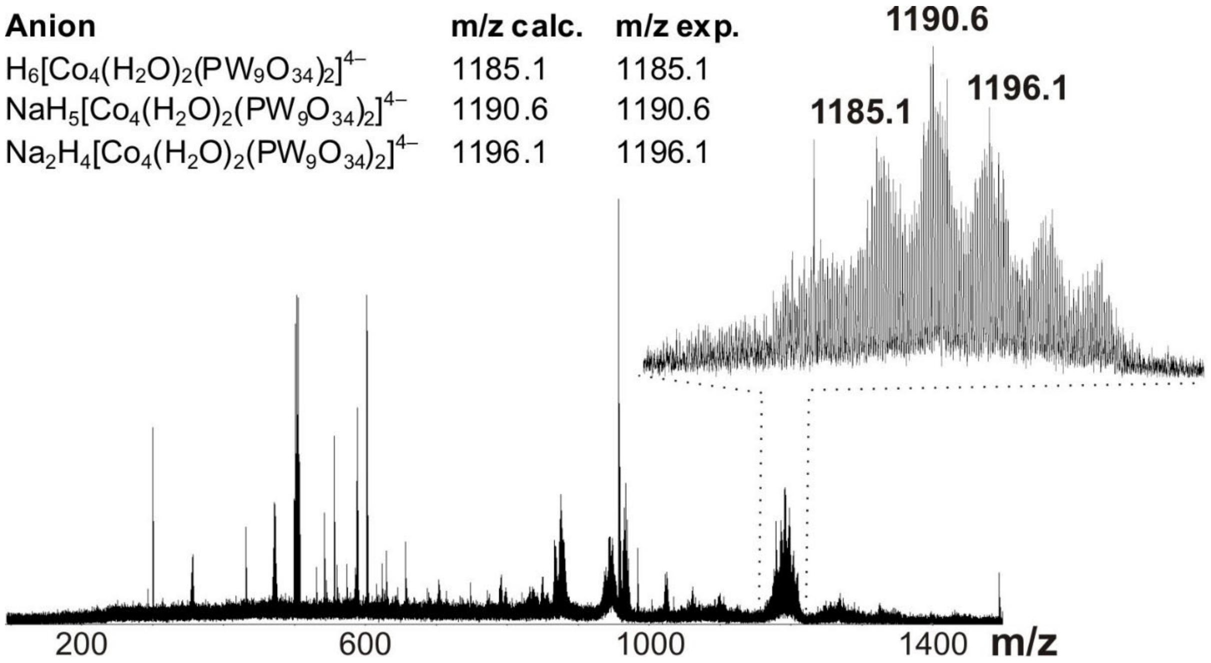

Figure S1. Negative ion-mode ESI-MS spectrum of **{$Co_4P_2W_{18}$}** in $H_2O/CH_3CN/MeOH$ mixture.

Table S2. Survey of existing cobalt-cubane containing germanotungstate crystal structures according to the *ICSD* database (February 2021).

POM	Investigated properties	Cluster-type and number of Co centers	Ref.
$[Co(H_2O)_3Ge_3W_{33}Co_3O_{114}]^{10-}$	Magnetism	Trimer with 6 Co^{2+}	[7]
$[Dy_{30}Co_8(OH)_{42}(H_2O)_{30}Ge_{12}W_{108}O_{408}]^{56-}$	Magnetism	Dodecamer with 8 Co^{2+}	[8]
$[Co_6(H_2O)_2Ge_3W_{24}O_{94}]^{20-}$	/	Dimer with 6 Co^{2+}	[9]
$[Co_4(H_2O)_2Ge_2W_{18}O_{72}]^{12-}$	Magnetism	Dimer with 4 Co^{2+}	[10]
$[Co_3W(H_2O)_2Ge_2W_{18}O_{72}]^{11-}$	Magnetism	Dimer with 3 Co^{2+}	[10]
$[Co_6(H_2O)_{12}Ge_2W_{18}O_{72}]^{8-}$	Electrochemical behavior	Dimer with 6 Co^{2+}	[11]
$[Co_4(H_2O)_2\ Ge_2W_{18}O_{72}]^{12-}$	Electrochemical behavior	Dimer with 4 Co^{2+}	[12]
$[Co_2(H_2O)_7Ge_2W_{18}O_{66}]^{12-}$	Electrochemical behavior	Dimer with 2 Co^{2+}	[12]
$[Co_{13}(OH)_4(H_2O)_2Ge_4W_{34}O_{126}]^{22-}$	Magnetism	Octamer with13 Co^{2+}	[13]
$[Co_{16}(OH)_{12}(PO_4)_4Ge_4W_{36}O_{136}]^{32-}$	WOC	Tetramer with 16 Co^{2+}	[14]
$[Co(H_2O)_3Ge_2W_{18}O_{66}]^{13-}$	Electrochemical behavior	Dimer with 1 Co^{2+}	[15]
$[Co_9(OH)_3(H_2O)_6(PO_4)_2Ge_3W_{27}O_{102}]^{21-}$	Magnetism/WOC	Trimer with 9 Co^{2+}	[9]
$[Co_4(Hdap)_2H_2Ge_2W_{18}O_{68}]^{8-}$ (dap = 1,2-diaminopropane)	Magnetism	Dimer with 4 Co^{2+}	[16]
$[Co_4(H_2O)_{14}Ge_4W_{36}O_{130}]^{20-}$	/	Tetramer with 4 Co^{2+}	[17]
$[Co_4(enH)_2Ge_2W_{18}O_{72}]^{10-}$ (en = ethylenediamine)	/	Dimer with 4 Co^{2+}	[18]

Table S3. Prominent representatives of cobalt-containing POMs splitting water photochemically in a basic environment within a $Ru(bpy)_3^{2+}$ (1 mM)/$S_2O_8^{2-}$(5 mM) sacrificial cycle according to *Scifinder* and *Web of Science* (February 2021). Note that various parameters such as the shape of the reaction vessel, light intensity, stirring rate as well as the ratio of gaseous head space to total volume render a direct comparison of the WOC performance difficult.[19]

POM	Conc. (µM)	Buffer and pH	TOF, s^{-1}	µmol O_2 (TON)	O_2 yield	Literature
$[Co_9(H_2O)_6(OH)_3(PW_9O_{34})_3]^{16-}$	6.6	20 mM phosphate buffer, pH 8	0.005	1.0 (10)	2.70%	[20]
$[Co_9(H_2O)_6(OH)_3(PW_9O_{34})_3]^{16-}$	16.33	20 mM phosphate buffer, pH 8	0.0045	1.3 (5.3)	3.50%	[20]
$[Co_6(H_2O)_{30}\{Co_9Cl_2(OH)_3(H_2O)_9(SiW_8O_{31})_3\}]^{5-}$	1.27	20 mM phosphate buffer, pH 8	0.042	2.0 (105)	5.30%	[20]
$[Co_6(H_2O)_{30}\{Co_9Cl_2(OH)_3(H_2O)_9(SiW_8O_{31})_3\}]^{5-}$	3.86	20 mM phosphate buffer, pH 8	0.021	3.1 (53)	8.30%	[20]
$[Co_6(H_2O)_{30}\{Co_9Cl_2(OH)_3(H_2O)_9(SiW_8O_{31})_3\}]^{5-}$	9.8	20 mM phosphate buffer, pH 8	0.019	4.1 (28)	10.9	[20]
$[\{Co_4(OH)_3PO_4\}_4(PW_9O_{34})_4]^{28-}$	3.62	20 mM phosphate buffer, pH 8	0.024	2.0 (37)	5.30%	[20]
$[\{Co_4(OH)_3PO_4\}_4(PW_9O_{34})_4]^{28-}$	9.19	20 mM phosphate buffer, pH 8	0.022	3.9 (28)	10.40%	[20]
$[\{Co_4(OH)_3(PO_4)\}_4(SiW_9O_{34})_4]^{32-}$	20	80 mM borate buffer, pH 9	0.053	9 (22.5)	18.10%	[21]
$[\{Co_4(OH)_3(PO_4)\}_4(GeW_9O_{34})_4]^{32-}$	20	80 mM borate buffer, pH 9	0.105	15.5 (38.75)	31.00%	[14]
$[\{Co_4(OH)_3(PO_4)\}_4(PW_9O_{34})_4]^{28-}$	20	80 mM borate buffer, pH 9	No data	8.7(20.25)	17.5%	[14]
$[\{Co_4(OH)_3(PO_4)\}_4(AsW_9O_{34})_4]^{28-}$	20	80 mM borate buffer, pH 9	No data	13.2 (33)	26.4%	[14]
$[Co_4(H_2O)_2(PW_9O_{34})_2]^{10-}$	5	80 mM borate buffer, pH 8	5	(224)	45.00%	[1c]
$[Co_4(H_2O)_2(VW_9O_{34})_2]^{10-}$	2	80 mM borate buffer, pH 9	1000	3 (35)	60%	[22]
$[\{Co_4(H_2O)_3(OH)\}(Si_2W_{19}O_{70})]^{11-}$	10	25 mM borate buffer, pH 9	0.100	4.8(80)	24%	[23]
$[Co_2(\beta\text{-}SiW_8O_{31})_2]^{16-}$	0.5	80 mM borate buffer, pH 9	7	(925)	18%	[24]

$[Co_2(\beta\text{-}SiW_8O_{31})_2]^{16-}$	1	80 mM borate buffer, pH 9	4	(674)	27%	[24]
$[Co_7As_6O_9(OH)_6(A\text{-}\alpha\text{-}SiW_9O_{34})_2]^{12-}$	1	80 mM borate buffer, pH 8	0.140	(115)	4.6%	[25]
$[Co_7As_6O_9(OH)_6(A\text{-}\alpha\text{-}SiW_9O_{34})_2]^{12-}$	20	80 mM borate buffer, pH 8	0.037	48.2	38.4%	[25]
$[Co(H_2O)_2(\gamma\text{-}SiW_{10}O_{35})_2]^{10-}$	5	80 mM borate buffer, pH 9	1.2	(186.9)	37.4	[26]
$[Co_8(OH)_6(H_2O)_2(CO_3)_3(A\text{-}\alpha\text{-}SiW_9O_{34})_2]^{16-}$	2	80 mM borate buffer, pH 9	10	(363)	29.1%	[27]
$[Co_8(OH)_6(H_2O)_2(CO_3)_3(A\text{-}\alpha\text{-}SiW_9O_{34})_2]^{16-}$	4	80 mM borate buffer, pH 9	10	(206)	16.4%	[27]
$[Co_4(H_2O)_{24}(B\text{-}\alpha\text{-}Bi_2W_{18}O_{60})]^{8-}$	0.5	80 mM borate buffer, pH 9	10	(1436)	28.7%	[28]
$[Co_4(H_2O)_{24}(B\text{-}\alpha\text{-}Bi_2W_{18}O_{60})]^{8}$	1	80 mM borate buffer, pH 9	5.6	(804)	32.2	[28]
$[Co_4(H_2O)_{24}(B\text{-}\alpha\text{-}Bi_2W_{18}O_{60})]^{8}$	2	80 mM borate buffer, pH 9	3.1	(545)	43.6	[28]
$[Co_4(H_2O)_{24}(B\text{-}\alpha\text{-}Bi_2W_{18}O_{60})]^{8}$	4	80 mM borate buffer, pH 9	1.5	(297)	47.5	[28]
$[Co^{II}Co^{III}(H_2O)W_{11}O_{39}]^{7-}$	1	80 mM borate buffer, pH 9	No data	6.5 (361)	15%	[29]
$[Co^{II}Co^{III}(H_2O)W_{11}O_{39}]^{7-}$	5	80 mM borate buffer, pH 9	0.5	9.0 (100)	20%	[29]
$[Co^{II}Co^{III}(H_2O)W_{11}O_{39}]^{7-}$	10	80 mM borate buffer, pH 9	No data	12.7 (71)	28%	[29]
$[Co^{II}Co^{III}(H_2O)W_{11}O_{39}]^{7-}$	15	80 mM borate buffer, pH 9	No data	13.7 (51)	30%	[29]
$[Co^{II}Co^{III}(H_2O)W_{11}O_{39}]^{7-}$	20	80 mM borate buffer, pH 9	No data	11.8 (33)	26%	[29]

3. IR-spectroscopy

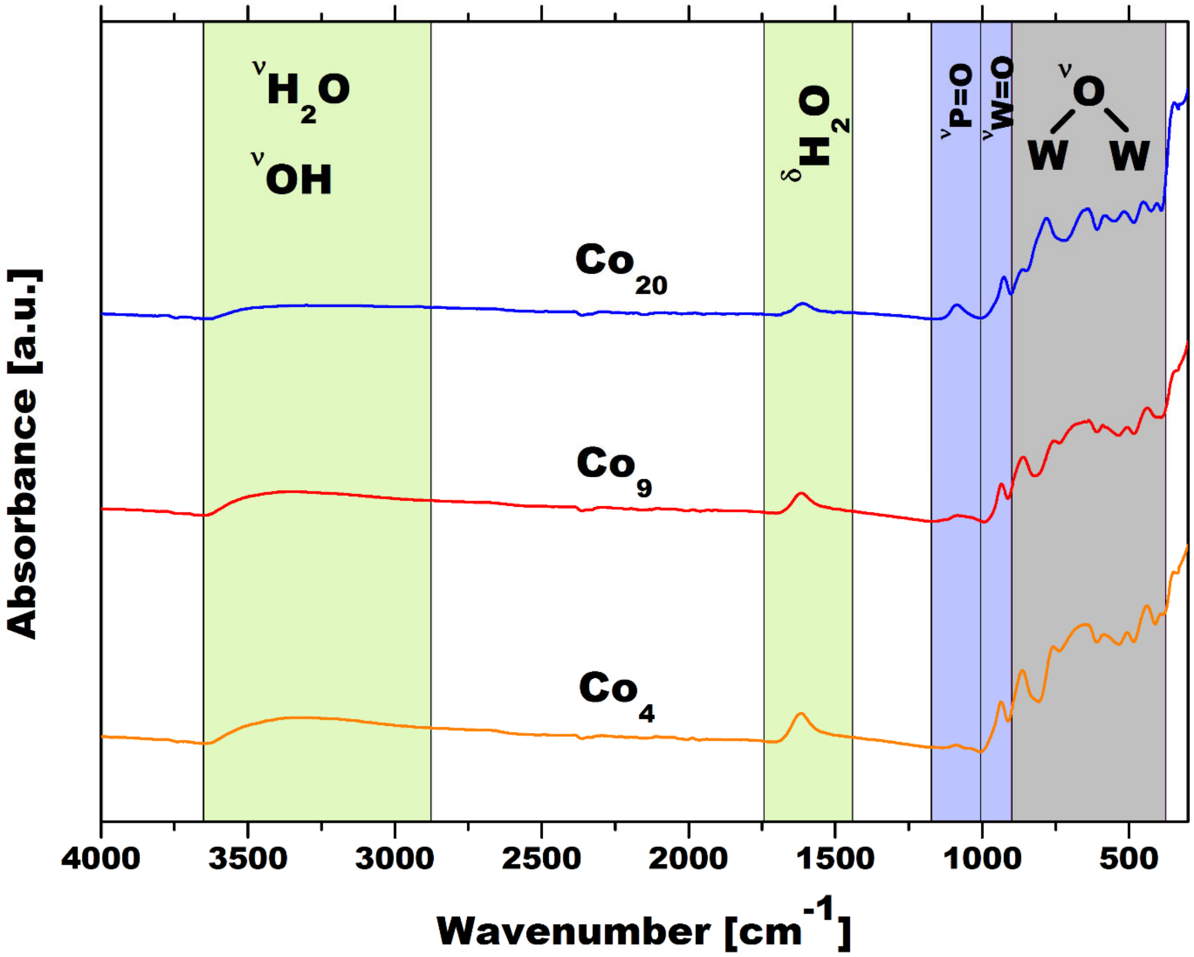

Figure S2. Superimposed FTIR spectra of **Co_4**, **Co_9** and **Co_{20}**. Both the absorption ranges of the W-O-W and W=O vibrations as well as those of water are highlighted in color.

4. Thermogravimetric Analysis

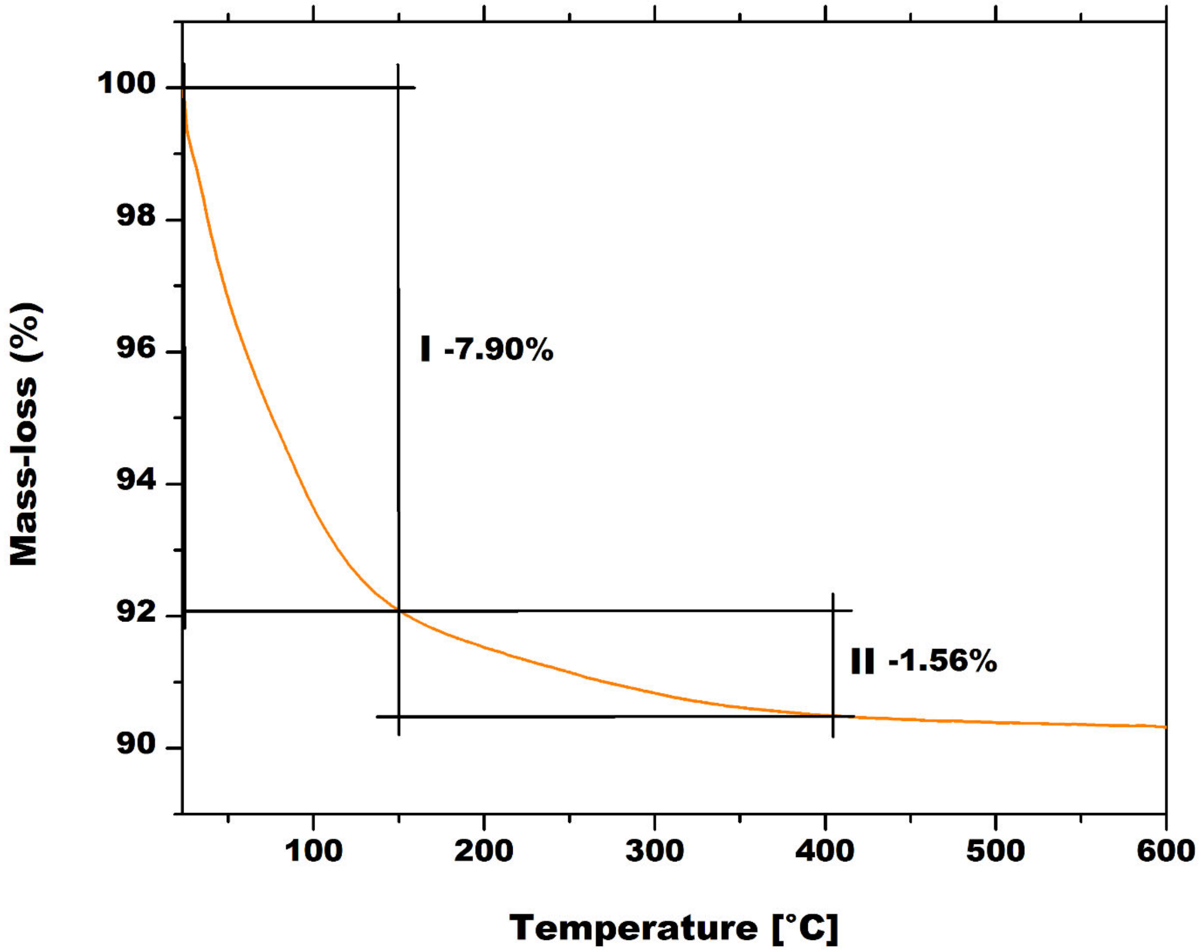

Figure S3. Thermogravimetric curve of **Co4** in the temperature region 25–600ºC with a heating rate of 5 ºC min^{-1}.

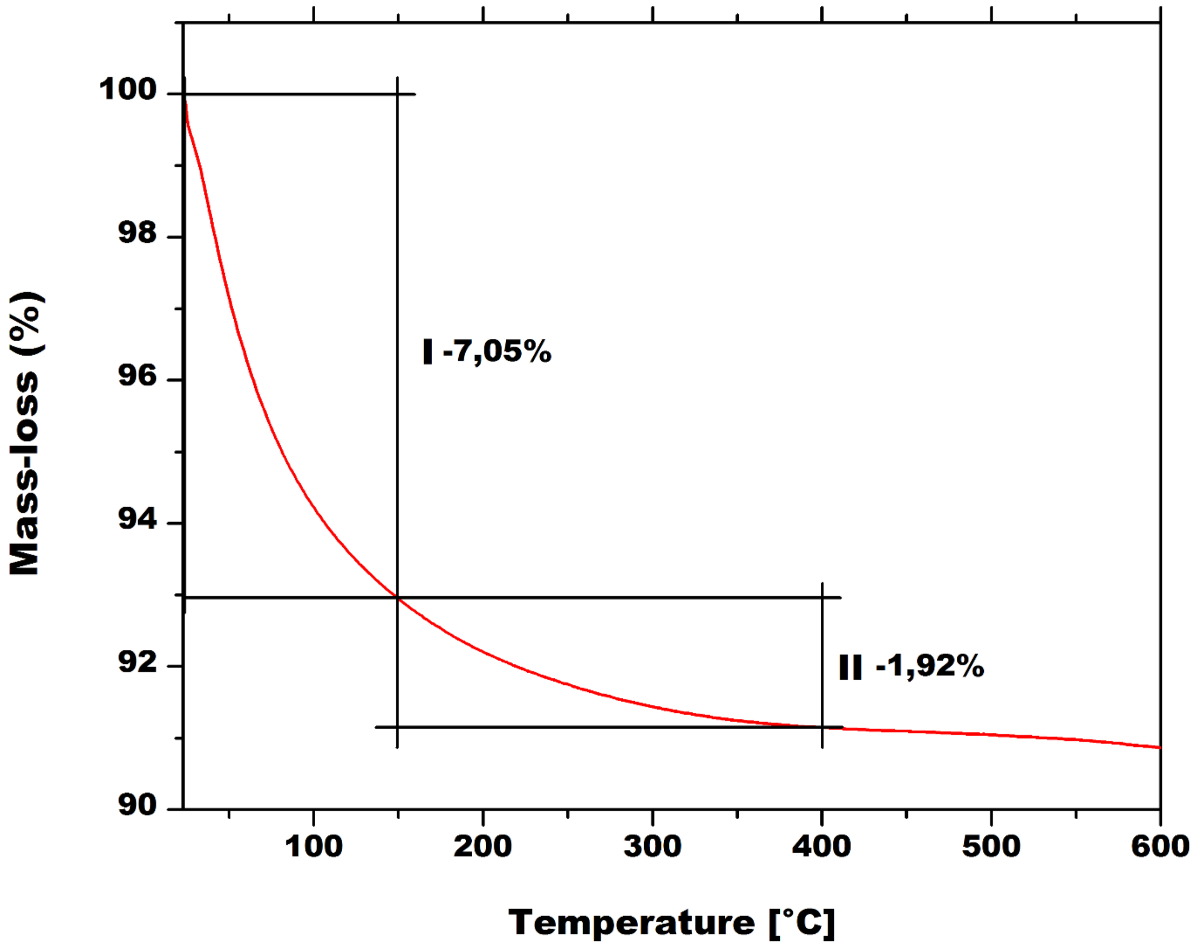

Figure S4. Thermogravimetric curve of **Co_9** in the temperature region 25–600ºC with a heating rate of 5 ºC min^{-1}.

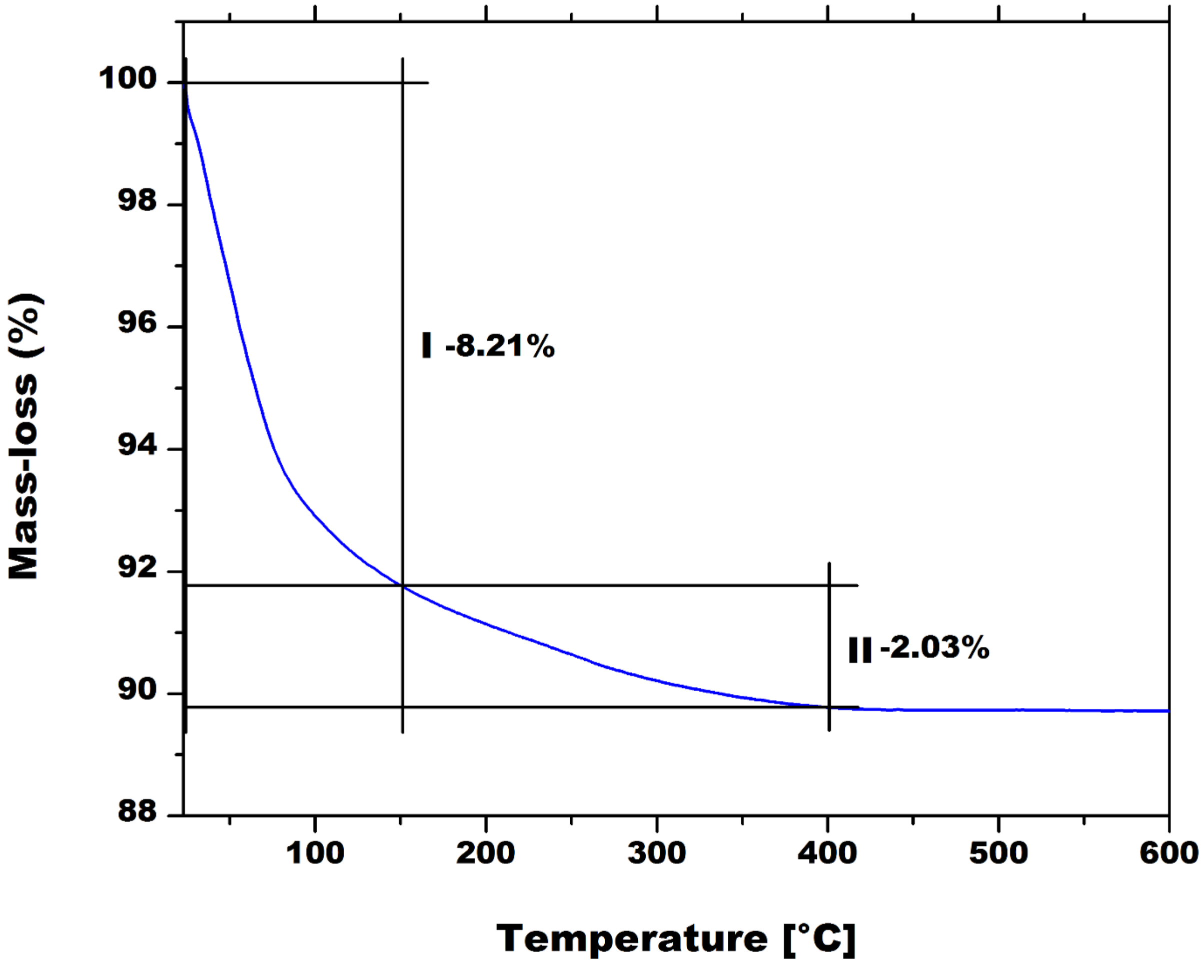

Figure S5. Thermogravimetric curve of **Co_{20}** in the temperature region 25–600ºC with a heating rate of 5 ºC min^{-1}.

Table S4. TGA results of **Co_4**, **Co_9**, and **Co_{20}**

	Step	T, °C	mass-loss, mg	mass-loss, %	number of H_2O corresponding to mass-loss
Co_4	I	23-150	0.74	7.9	33
	II	150-400	0.15	1.6	7
Co_9	I	23-150	1.41	1.4	39
	II	150-400	0.38	0.4	10
C_{20}	I	23-150	1.52	1.5	63
	II	150-400	0.38	0.4	16

5. Single-Crystal X-ray Diffraction

Table S5. Sample and crystal data of **Co_4**

**Chemical formula**	$Co_4Ge_4Na_8O_{136}PW_{24}$	**Crystal system**	monoclinic	
**Formula weight [g/mol]**	7329.37	**Space group**	$P2_1/c$	
**Temperature [K]**	200	**Z**	4	
**Measurement method**	φ and omega ω sans	**Volume [Å^3]**	13639(2)	
**Radiation (Wavelength [Å])**	MoKα (λ = 0.71073)	**Unit cell dimensions [Å] and [°]**	17.6471(17)	90.0
**Crystal size / [mm^3]**	0.4 × 0.15 × 0.1		22.332(2)	95.876(5)
**Crystal habit**	clear pink plates		34.790(3)	90.0
**Density (calculated) / [g/cm^3]**	3.569	**Absorption coefficient / [mm^{-1}]**	21.636	
**Abs. correction Tmin**	0.3496	**Abs. correction Tmax**	0.7461	
**Abs. correction type**	multi-scan	**F(000) [e$^-$]**	12812	

Table S6. Data collection and structure refinement of **Co_4**

**Index ranges**	-21 ≤ h ≤ 21, -26 ≤ k ≤ 26, -41 ≤ l ≤ 41	**2 Theta range for data collection [°]**	2.17 to 50.7	
**Reflections number**	250413	**Data / restraints / parameters**	24078/84/1576	
**Refinement method**	Least - squares	**Final R indices**	all data	R_1 = 0.0642, wR_2 = 0.1323
**Function minimized**	$\Sigma\ w(F_o^2 - F_c^2)^2$		I>2σ(I)	R_1 = 0.0525, wR_2 = 0.1244
**Goodness-of-fit on F^2**	1.075	**Weighting scheme**	$w=1/[\sigma^2(F_o^2)+(0.0336P)^2+1279.8711P]$	
**Largest diff. peak and hole [e Å^{-3}]**	6.69/-2.82		where $P=(F_o^2+2F_c^2)/3$	

Table S7. Sample and crystal data of **Co_9**

**Chemical formula**	$Co_9Ge_3K_{11.5}Na_4O_{158}PW_{30}$	**Crystal system**	triclinic
**Formula weight [g/mol]**	9364.22	**Space group**	$P\bar{1}$
**Temperature [K]**	200	**Z**	2
**Measurement method**	φ and omega ω sans	**Volume [Å^3]**	7529(3)

**Radiation (Wavelength [Å])**	MoKα (λ = 0.71073)	**Unit cell dimensions [Å] and [°]**	19.896(5)	101.722(2)
**Crystal size / [mm³]**	0.225 × 0.2 × 0.075		20.634(5)	114.729(2)
**Crystal habit**	clear pink block		21.094(5)	95.845(2)
**Density (calculated) / [g/cm³]**	4.130	**Absorption coefficient / [mm⁻¹]**	24.818	
**Abs. correction Tmin**	0.2420	**Abs. correction Tmax**	0.7451	
**Abs. correction type**	multi-scan	**F(000) [e⁻]**	8201	

Table S8. Data collection and structure refinement of **Co_9**

**Index ranges**	-23 ≤ h ≤ 23, -23 ≤ k ≤ 23, -24 ≤ l ≤ 24	**2 Theta range for data collection [°]**	2.304 to 48.814	
**Reflections number**	127468	**Data / restraints / parameters**	24757/36/1896	
**Refinement method**	Least - squares	**Final R indices**	all data	R_1 = 0.0683, wR_2 = 0.1228
**Function minimized**	Σ w(F_o^2 - F_c^2)^2		I>2σ(I)	R_1 = 0.0514, wR_2 = 0.1153
**Goodness-of-fit on F^2**	1.093	**Weighting scheme**	w=1/[σ^2(F_o^2)+(0.0185P)^2+ 651.3266P]	
**Largest diff. peak and hole [e Å⁻³]**	5.19/-2.44		where P=(F_o^2+2F_c^2)/3	

Table S9. Sample and crystal data of **Co_{20}**

**Chemical formula**	$Co_{23}Ge_4K_{11.59}Na_{11.02}O_{253}P_4W_{36}$	**Crystal system**	monoclinic	
**Formula weight [g/mol]**	13142.79	**Space group**	*C*2/*c*	
**Temperature [K]**	200	**Z**	4	
**Measurement method**	φ and omega ω sans	**Volume [Å³]**	23316(7)	
**Radiation (Wavelength [Å])**	MoKα (λ = 0.71073)	**Unit cell dimensions [Å] and [°]**	41.602(6)	90
**Crystal size / [mm³]**	0.5 × 0.5 × 0.5		17.892(3)	122.8810(10)
**Crystal habit**	clear pink rod		37.300(8)	90
**Density (calculated) / [g/cm³]**	3.744	**Absorption coefficient / [mm⁻¹]**	20.152	

**Abs. correction Tmin**	0.0034	**Abs. correction Tmax**	0.7461
**Abs. correction type**	multi-scan	**F(000) [e⁻]**	23354.0

Table S10. Data collection and structure refinement of **Co_{20}**

**Index ranges**	$-52 \le h \le 52$, $-21 \le k \le 19$, $-46 \le l \le 46$	**2 Theta range for data collection [°]**	5.712 to 52.744	
**Reflections number**	85895	**Data / restraints / parameters**	23351/48/1541	
**Refinement method**	Least - squares	**Final R indices**	all data	R_1 = 0.0455, wR_2 = 0.0854
**Function minimized**	$\Sigma\, w(F_o^2 - F_c^2)^2$		$I>2\sigma(I)$	R_1 = 0.0356, wR_2 = 0.0809
**Goodness-of-fit on F^2**	1.047	**Weighting scheme**	$w=1/[\sigma^2(F_o^2)+(0.0256P)^2+1120.2599P]$	
**Largest diff. peak and hole [e Å^{-3}]**	6.58/-4.32		where $P=(F_o^2+2F_c^2)/3$	

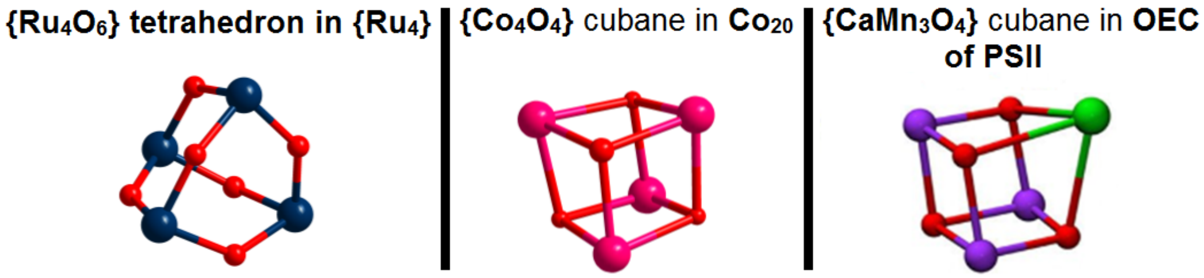

Figure S6. Ball-and-stick representation of {Ru_4O_6} in **{Ru_4}** (left: Ru, dark blue; O, red), {Co_4O_4} in **Co_{20}** (middle: Co, pink; O, red) and **{Mn_3CaO_4}** in OEC of PSII (right: Mn, violet; Ca, green; O, red). The tetrahedral arrangement of the catalytic center in **{Ru_4}** is shown on the left. In the middle the cubic center in **Co_{20}**, is depicted, which shows a strong geometrical similarity to the cubic catalytic center in OEC of PSII. The {Co_4O_4} cubane and the {Mn_3CaO_4} cubane of the OEC in PSII exhibit similar metal-metal (Co-Co 3.2 Å, Mn-Mn 2.8-3.3 Å and Ca-Mn 3.3-3.5 Å) and metal-oxygen bond lengths (Co-O 2.1Å, Mn-O 1.8-2.1 Å and Ca-O 2.4-2.5). However, there is a noticeable difference in the bond length to a single oxygen in the cubic arrangement in the OEC, where the bond lengths are slightly larger (Ca-O 2.7 Å and Mn-O 2.4-2.6 Å).

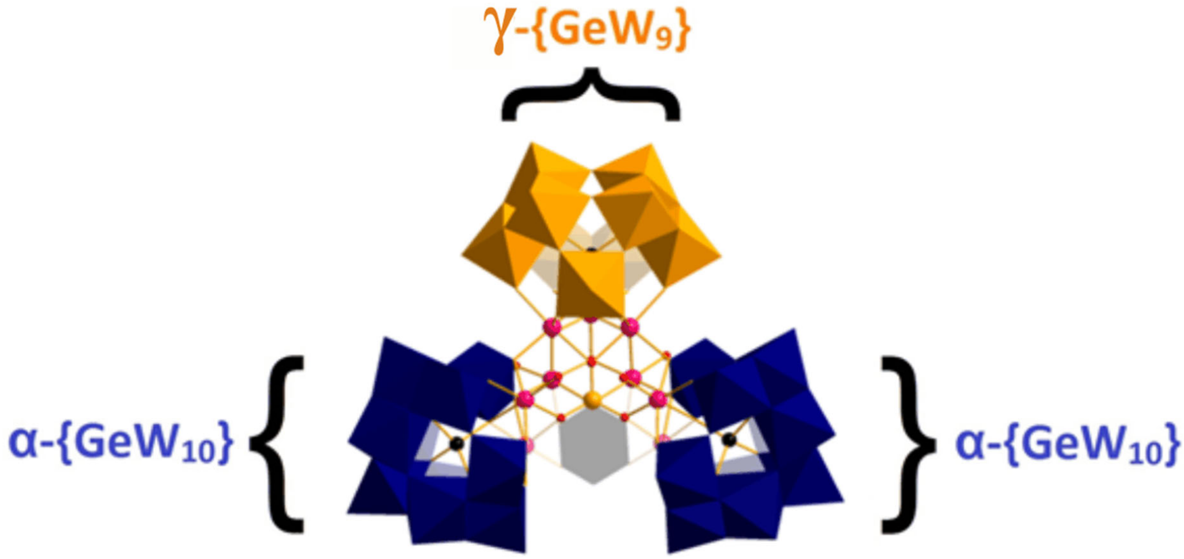

Figure S7. Combined polyhedral and ball-and-stick representation of $\mathbf{Co_9}$ with the labeled individual building blocks in orange and blue. $\mathbf{Co_9}$ crystallizes in space group P-1 and its scaffold is a dimeric assembly of α-{Co_2GeW_{10}} and a monomeric assembly of β-{Co_3GeW_9} Keggin units exhibiting S2 symmetry. Between both α-{Co_2GeW_{10}} Keggins, an {WO_6} octahedron is encapsulated, which is connected through four μ_2-oxygen atoms with cobalt ions and one μ_4-oxygen atom with {PO_4}. The cobalt-oxo core consists of nine cobalt ions with distorted octahedral coordination environment. According to BVS calculations (**Table S11**) all cobalt ions are divalent and their terminal oxygens deprotonated, suggesting water ligands. Furthermore, several bridging oxygens are monoprotonated. These are two μ_2- and three μ_3-oxygens in the backbone of the cobalt-oxo core. {WO_6} octahedra, grey; Co, pink; Ge, black; P, depicted as P; O, red.

6. Bond-Valence Summation (BVS) Analysis

Table S11. Selected bond valence sum (BVS) values for **Co_4**, **Co_9** and **Co_{20}**.

POM	Atom sort	Atom center	BVS Value	Assignment
**Co_4**	Co	Co1<br>Co2<br>Co3<br>Co4	2.00<br>1.96<br>1.99<br>2.00	2+<br>2+<br>2+<br>2+
	μ-O (Ge-O)	O17<br>O22	0.47<br>0.39	OH_2<br>OH_2
**POM**	**Atom sort**	**Atom center**	**BVS Value**	**Assignment**
**Co_9**	Co	Co1<br>Co2<br>Co3<br>Co4<br>Co5<br>Co6<br>Co7<br>Co8<br>Co9	2.01<br>1.98<br>2.03<br>2.03<br>2.02<br>1.80<br>1.84<br>1.97<br>1.88	2+<br>2+<br>2+<br>2+<br>2+<br>2+<br>2+<br>2+<br>2+
	μ-O (Co-O)	O119<br>O121<br>O118<br>O123	0.35<br>0.23<br>0.35<br>0.34	OH_2<br>OH_2<br>OH_2<br>OH_2
	μ_2-O (Co-O-W)	O115<br>O116	0.99<br>1.14	OH^-<br>OH^-
	μ_3-O (3Co-O)	O120<br>O122<br>O124	1.01<br>1.07<br>1.00	OH^-<br>OH^-<br>OH^-
**POM**	**Atom sort**	**Atom center**	**BVS Value**	**Assignment**
**Co_{20}**	Co	Co1<br>Co2<br>Co3<br>Co4<br>Co5<br>Co6<br>Co7<br>Co8<br>Co9<br>Co11	1.88<br>1.91<br>1.95<br>1.90<br>1.89<br>1.94<br>1.93<br>1.94<br>1.97<br>2.08	2+<br>2+<br>2+<br>2+<br>2+<br>2+<br>2+<br>2+<br>2+<br>2+
	μ-O (Co-O)	O94<br>O93<br>O95<br>O97<br>O79<br>O80<br>O77<br>O78<br>O96	0.36<br>0.31<br>0.35<br>0.36<br>0.41<br>0.32<br>0.27<br>0.33<br>0.34	OH_2<br>OH_2<br>OH_2<br>OH_2<br>OH_2<br>OH_2<br>OH_2<br>OH_2<br>OH_2
	μ_2-O (Co-O-P)	O6	1.19	OH^-
	μ_3-O (3Co-O)	O81<br>O86<br>O82<br>O85<br>O84<br>O83	1.02<br>1.05<br>1.05<br>1.03<br>1.04<br>1.04	OH^-<br>OH^-<br>OH^-<br>OH^-<br>OH^-<br>OH^-

7. Powder X-ray Diffraction (PXRD)

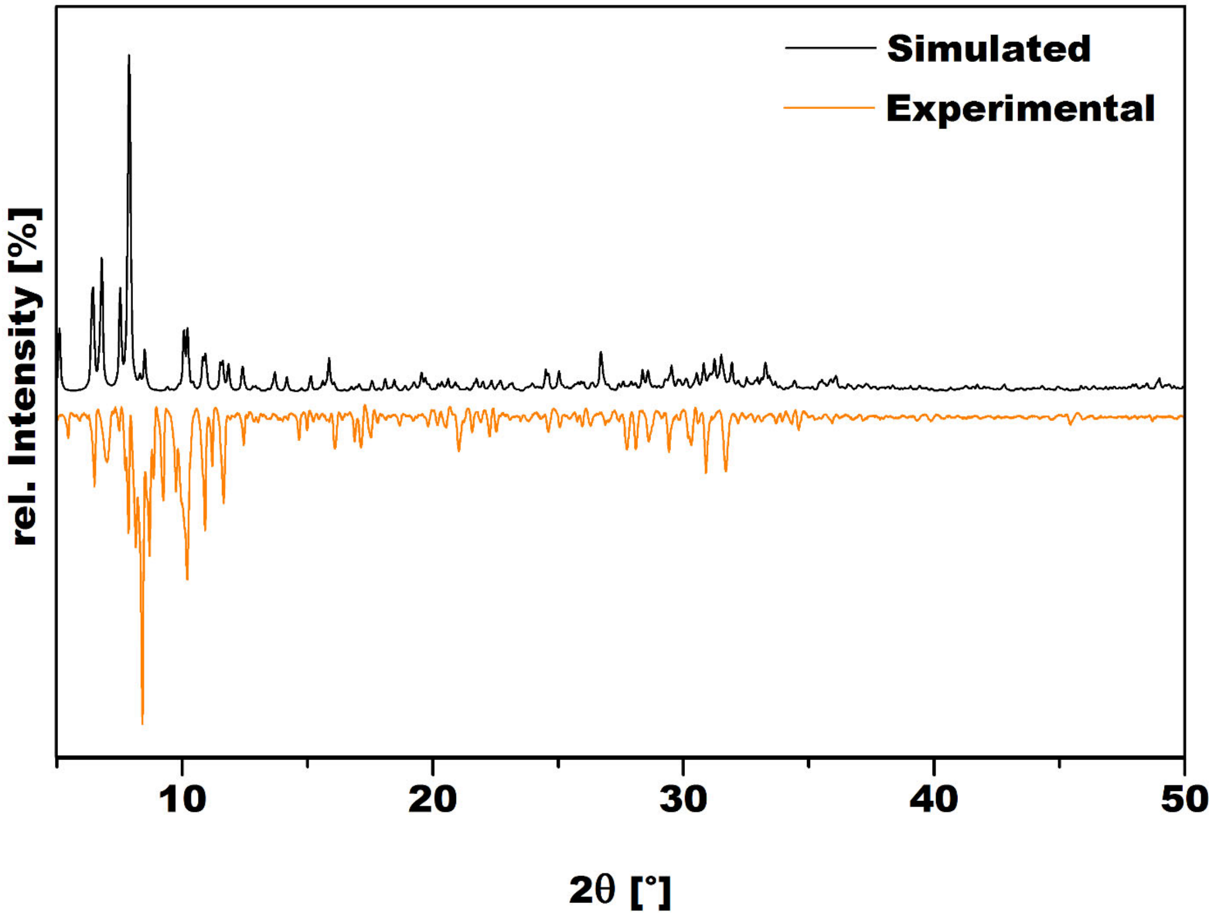

Figure S8. Comparison of the experimental and simulated PXRD pattern of **Co_4**. Note that differences between the simulated and the experimental PXRD patterns may be due to factors such as scanning speed, preferred orientation, and efflorescence of the crystals, which lose solvent molecules further leading to the collapse of the lattice.

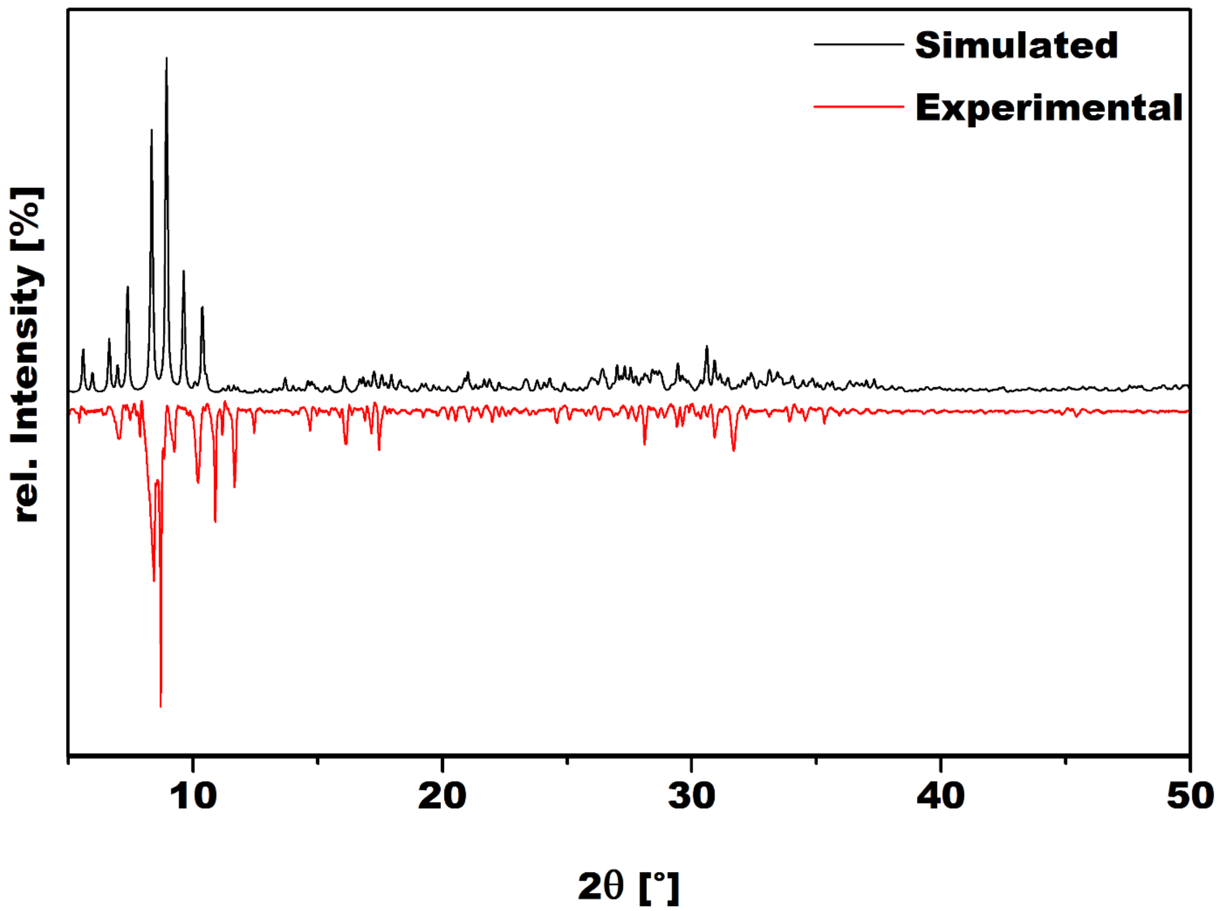

Figure S9. Comparison of the experimental and simulated PXRD pattern of $\mathbf{Co_9}$. Note that differences between the simulated and the experimental PXRD patterns may be due to factors such as scanning speed, preferred orientation, and efflorescence of the crystals, which lose solvent molecules further leading to the collapse of the lattice.

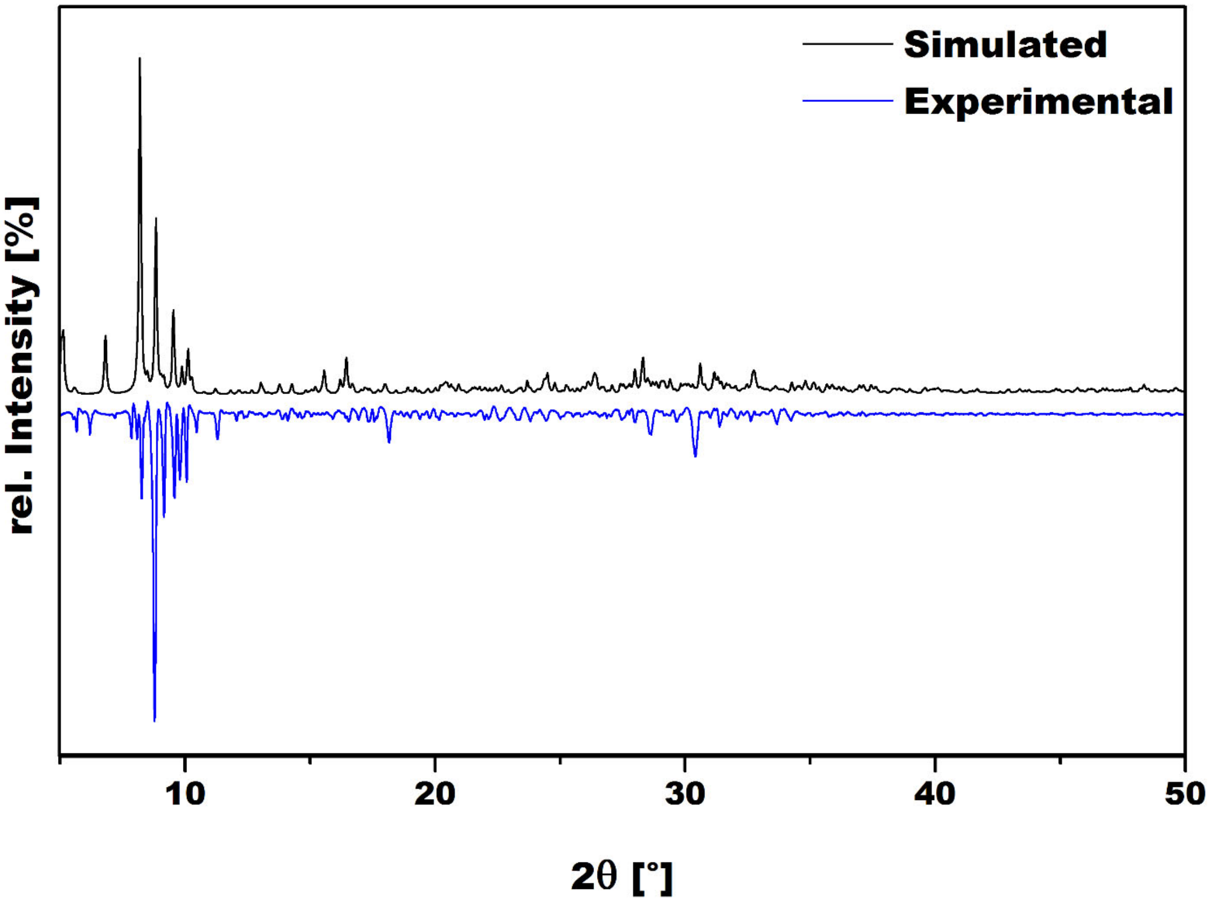

Figure S10. Comparison of the experimental and simulated PXRD pattern of **Co_{20}**. Note that differences between the simulated and the experimental PXRD patterns may be due to factors such as scanning speed, preferred orientation, and efflorescence of the crystals, which lose solvent molecules further leading to the collapse of the lattice.

8. Optical transitions

8.1. Estimation of E_g using diffuse reflectance spectroscopy (DRS)

To complement the computationally determined band gap trend for **Co_4** and **Co_9**, diffuse reflectance spectroscopic (DRS) measurements in the range from 200 to 1000 nm were performed on powdered samples of **Co_4** and **Co_9** (**Figures S11, S12**). The optical transitions of **Co_4** and **Co_9** could be estimated by using the Tauc plot against the energy E [eV] derived from the DRS spectra by applying **Equations S1 and S2** and determining the intersection point between the energy axis and the line extrapolated from the linear portion of the absorption edge (**Figures S13, S14**). The estimated optical transition values reveal the same trend E_g(**Co_9**) < E_g (**Co_4**) as observed from the DFT calculations thereby further supporting the experimental and computational findings of the WOC studies.

$F(R_\infty) = \frac{K}{S} = \frac{(1-R_\infty)^2}{2R_\infty}$, where K is the absorption coefficient,

S is the scattering factor and R is the reflectance [%]

obtained from the DRS spectrum (**Equation S1**)

$(\propto h\nu)^2 = (F(R_\infty) \times E)^{1/2}$ where $E = \frac{1239.7}{\lambda}$, $\lambda = \frac{1}{\nu}$, with λ being the corresponding x-axis value (nm) in the DRS spectrum (**Equation S2**)

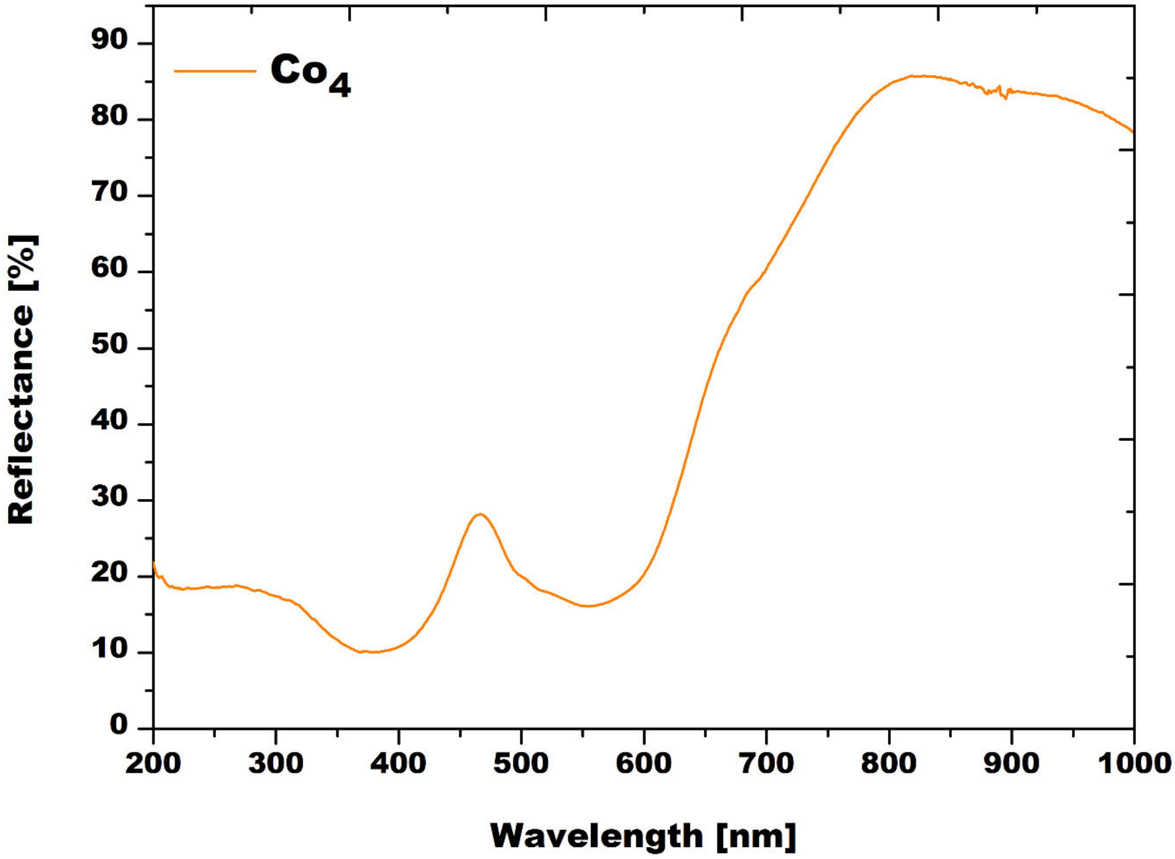

Figure S11. Diffuse reflectance spectrum of **Co_4**.

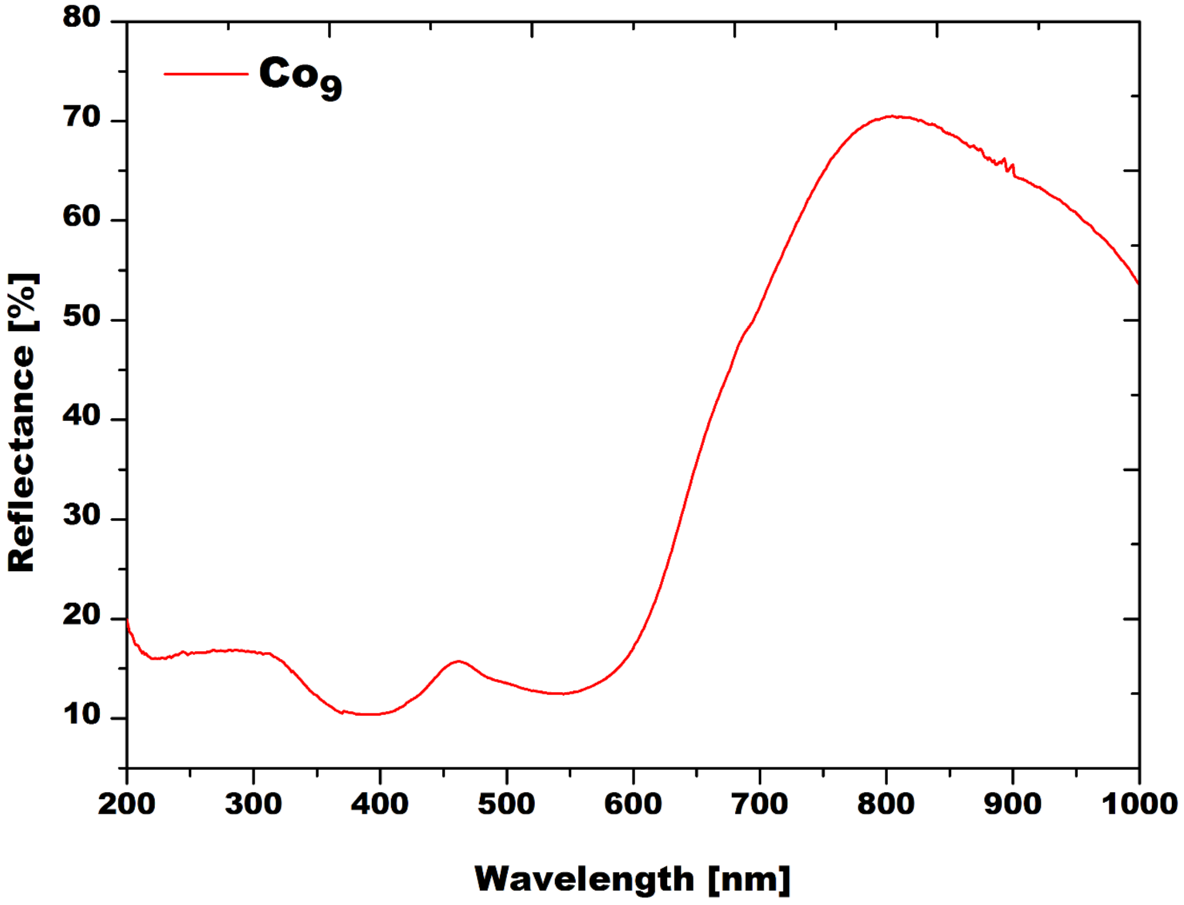

Figure S12. Diffuse reflectance spectrum of **Co_9**.

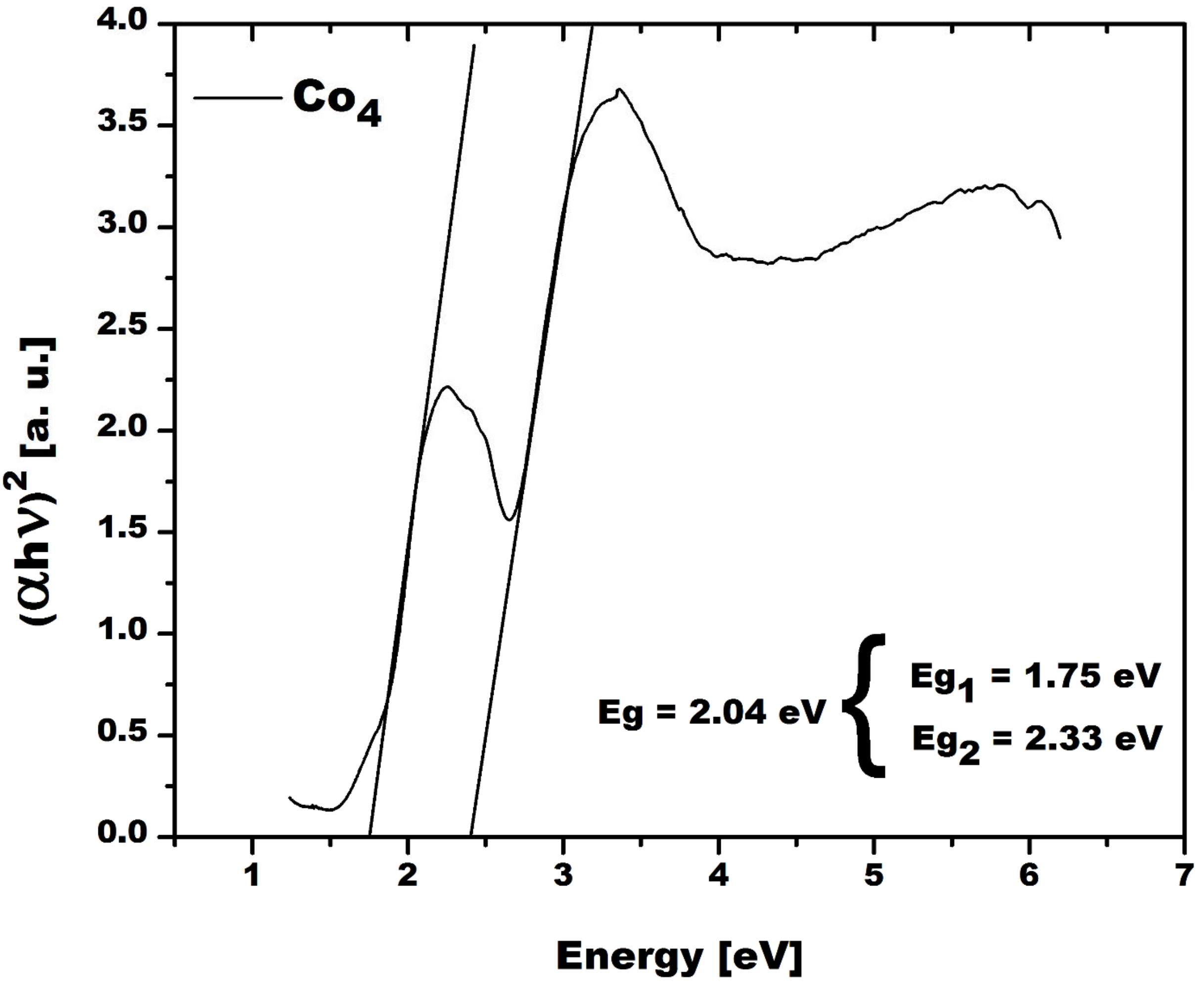

Figure S13. Tauc plot obtained from the diffuse reflectance spectrum of **Co_4** showing a direct (E_{g1} = 1.75 eV) and an indirect HOMO-LUMO gap value (E_{g2} = 2.33 eV) which may be attributed to the contribution of the phonons in the case of indirect transitions. [[30]] The HOMO-LUMO gap value E_g = 2.04 eV is obtained as an average value between E_{g1} and E_{g2}.

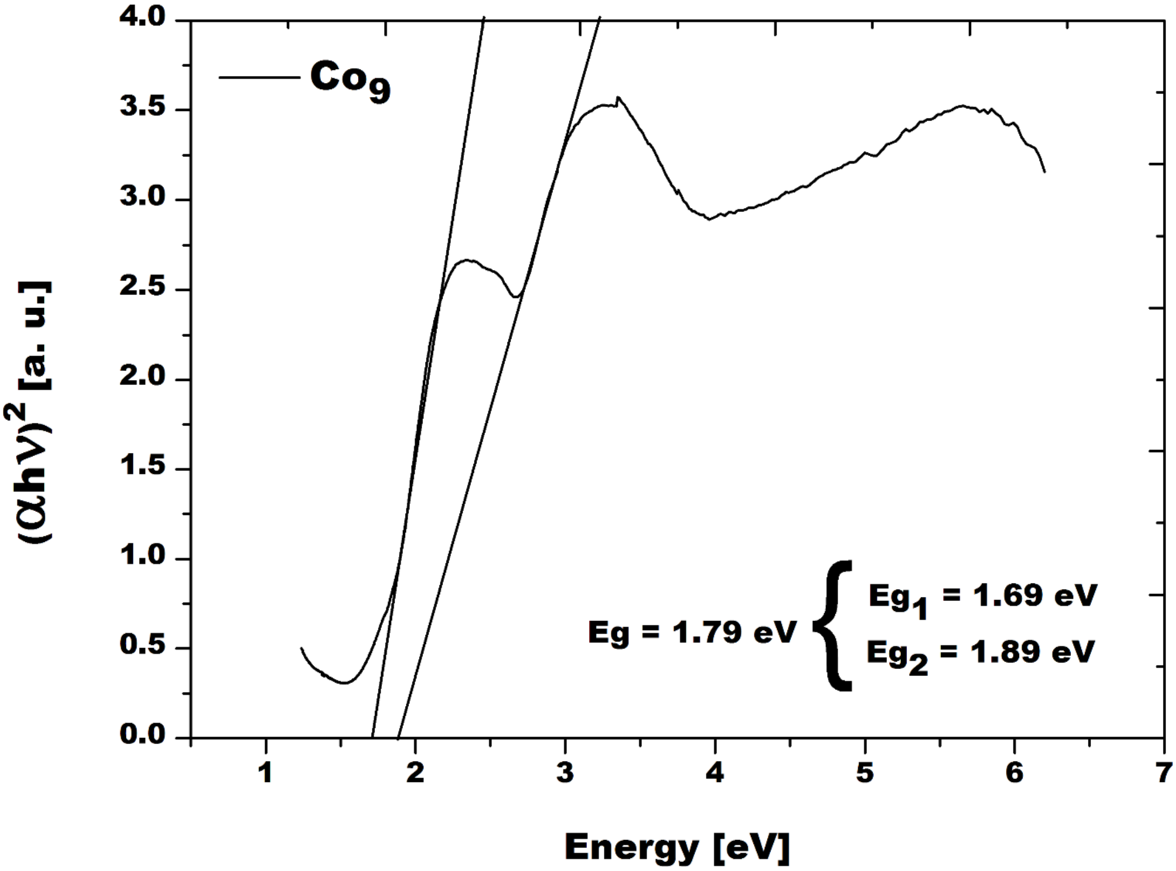

Figure S14. Tauc plot obtained from the diffuse reflectance spectrum of $\mathbf{Co_9}$ showing a direct (Eg_1 = 1.69 eV) and an indirect HOMO-LUMO gap value (E_{g2} = 1.89 eV) which may be attributed to the contribution of the phonons in the case of indirect transitions.[30] The HOMO-LUMO gap value E_g = 1.79 eV is obtained as an average value between E_{g1} and E_{g2}.

8.2. Estimation of band gap position using cyclic voltammetry (CV)

Considering that the LUMOs of POMs are formally a nonbonding combination of symmetry-adapted d_{xy} like orbits centering on the metal (W) atoms,[31] the LUMO levels could be estimated by finding out the applied onset potential for the first reduction of **Co_4** and **Co_9**,[32] and the HOMOs could be calculated according to **Equation S3**:

$$E[LUMO(vs\ Vacuum)] = E[HOMO(vs\ Vacuum)] + E_g \quad \textbf{(Equation S3)}$$

Taking into account that the reduction potentials of POMs in the cyclic voltammograms are dependent on the applied test environment, all the electrochemical experiments were carried out in borate buffer [80 mM], pH = 8.0, to eliminate the significant influence on the electrochemical response as far as possible. Based on this, the ground and excited energy levels of **Co_4** and **Co_9** could be determined and calculated (**Figures S15, S16**). By applying **Eq. 3**, estimated HOMO levels of -2.52 V (**Co_4**) and -2.04 V (**Co_9**) were obtained, thereby suggesting that the estimated HOMO levels of **Co_9** lie higher in energy than the ones of **Co_4** which is in accordance with the computational studies.

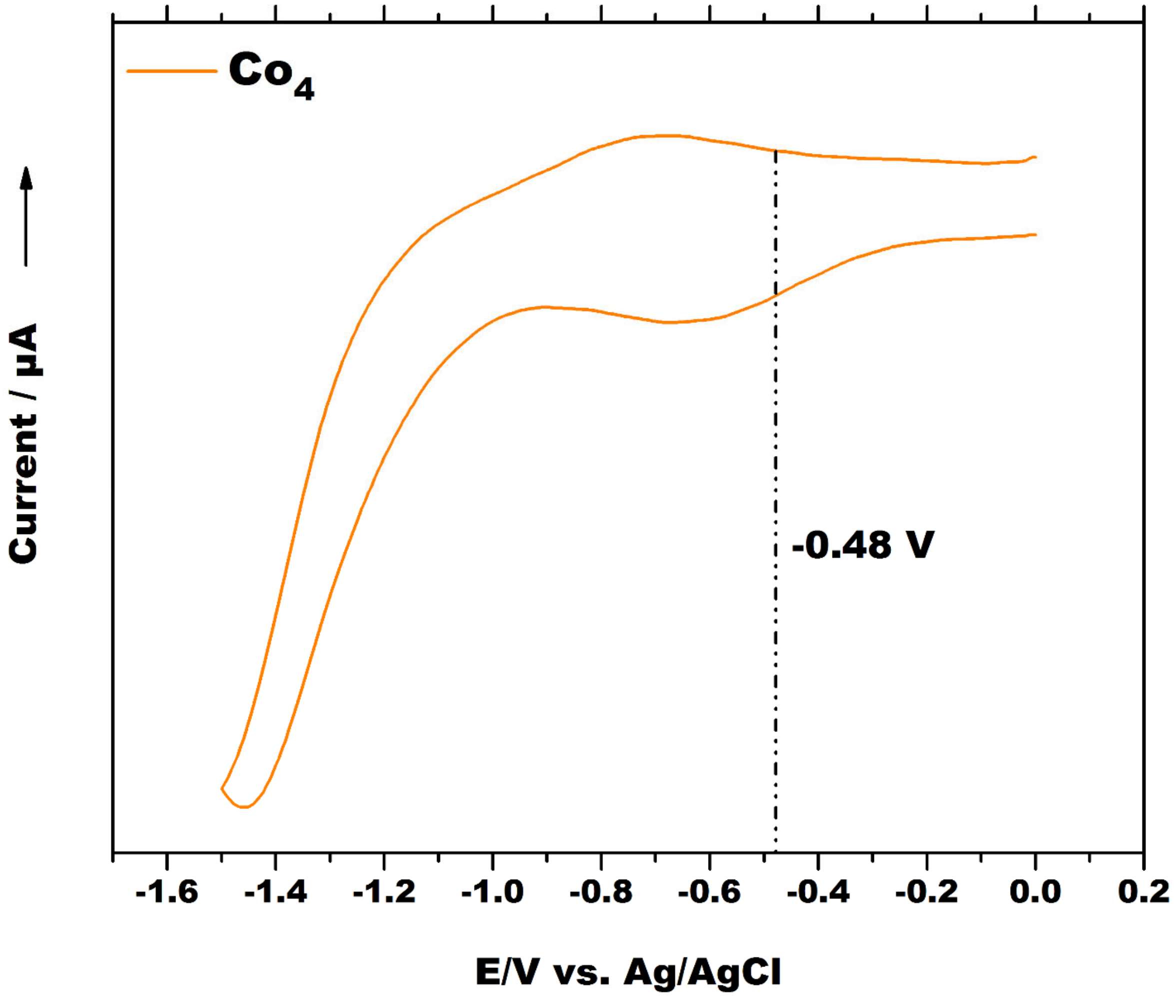

Figure S15. Cathodic range of the cyclic voltammogram of $\mathbf{Co_4}$ in borate buffer [80 mM], pH = 8.0 at a 100 mV s^{-1} scan rate. The intersection point of the dotted line and the X axis corresponds to the onset reduction potential of $\mathbf{Co_4}$.

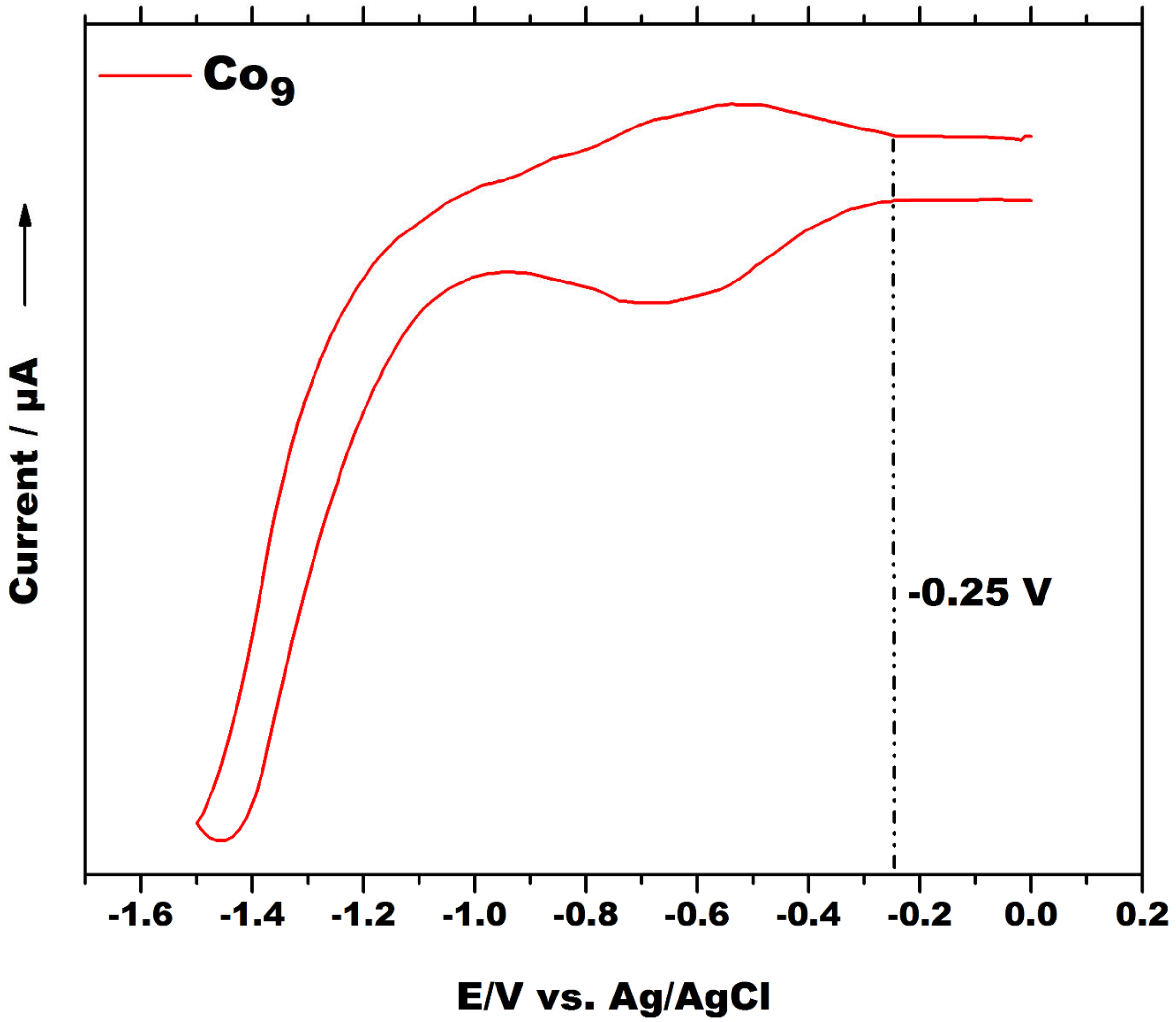

Figure S16. Cathodic range of the cyclic voltammogram of **Co_9** in borate buffer [80 mM], pH = 8.0 at a 100 mV s^{-1} scan rate. The intersection point of the dotted line and the X axis corresponds to the onset reduction potential of **Co_9**.

9. Pre-catalytic studies

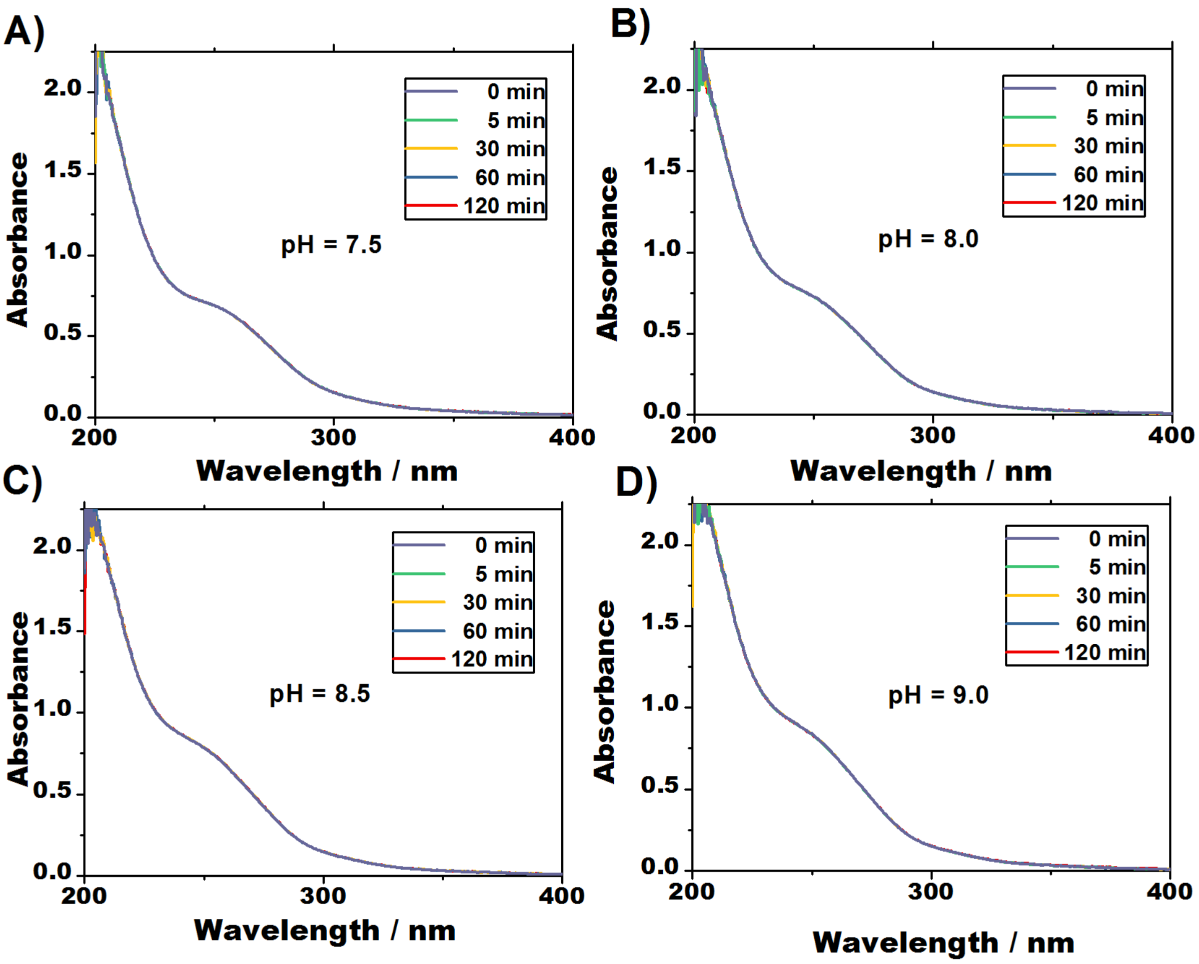

Figure S17. UV-vis spectra of **Co_4** (10.5 μM) at pH **A**) 7.5, **B)** 8.0, **C)** 8.5 and **D)** 9.0 in 80 mM sodium borate buffer are characterized by an absorption maximum ~205 nm with a shoulder at ~250 nm, which are attributed to the $p_{\pi}(O_t)\rightarrow d_{\pi^*}(W)$ ligand-to-metal charge-transfer transitions.

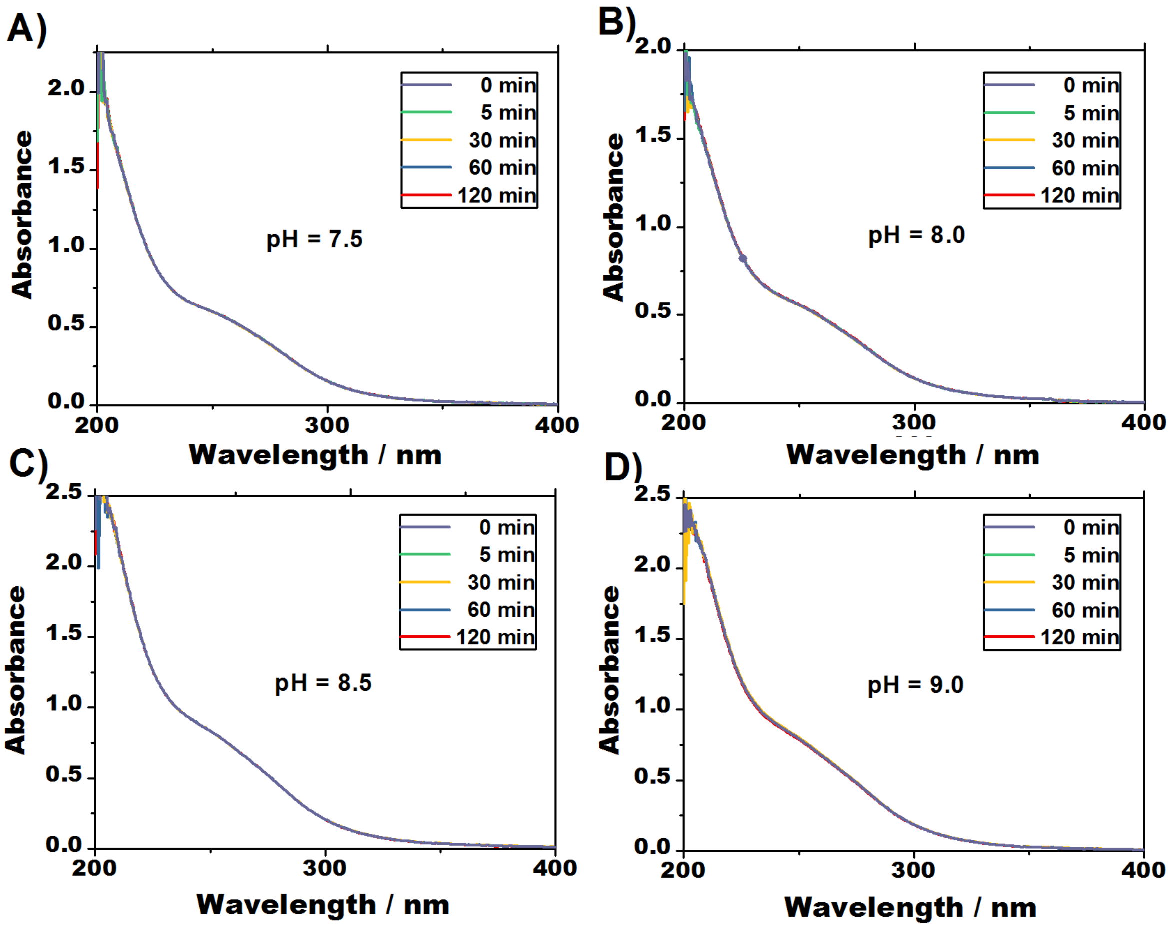

Figure S18. UV-vis spectra of **Co₉** (6.7 μM) at pH **A)** 7.5, **B)** 8.0, **C)** 8.5 and **D)** 9.0 in 80 mM sodium borate buffer are characterized by an absorption maximum ~205 nm with a shoulder at ~250 nm, which are attributed to the $p_{\pi}(O_t) \rightarrow d_{\pi^*}(W)$ ligand-to-metal charge-transfer transitions.

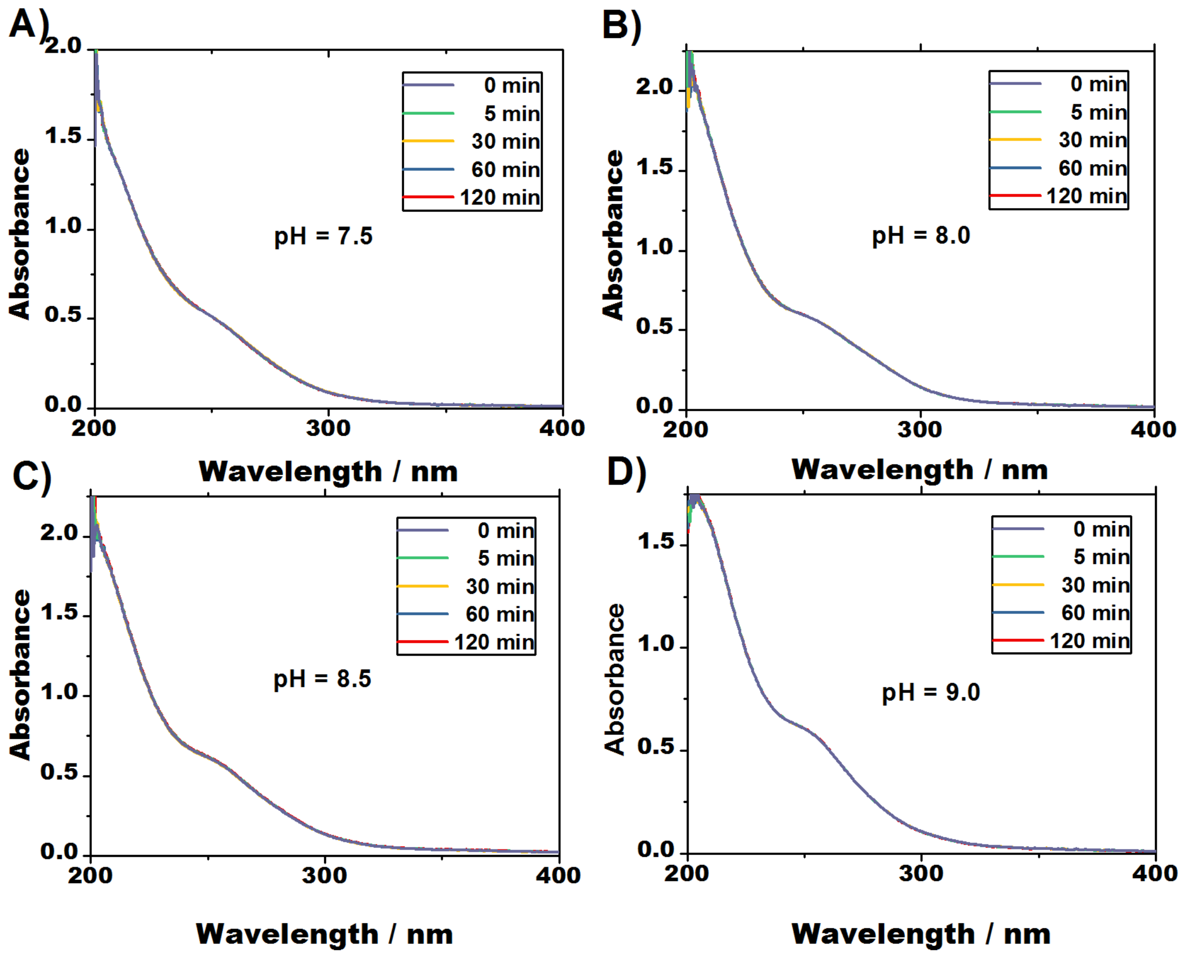

Figure S19. UV-vis spectra of **Co_{20}** (6.3 μM) at pH **A)** 7.5, **B)** 8.0, **C)** 8.5 and **D)** 9.0 in 80 mM sodium borate buffer are characterized by an absorption maximum ~205 nm with a shoulder at ~250 nm, which are attributed to the $p_{\pi}(O_t) \rightarrow d_{\pi^*}(W)$ ligand-to-metal charge-transfer transitions.

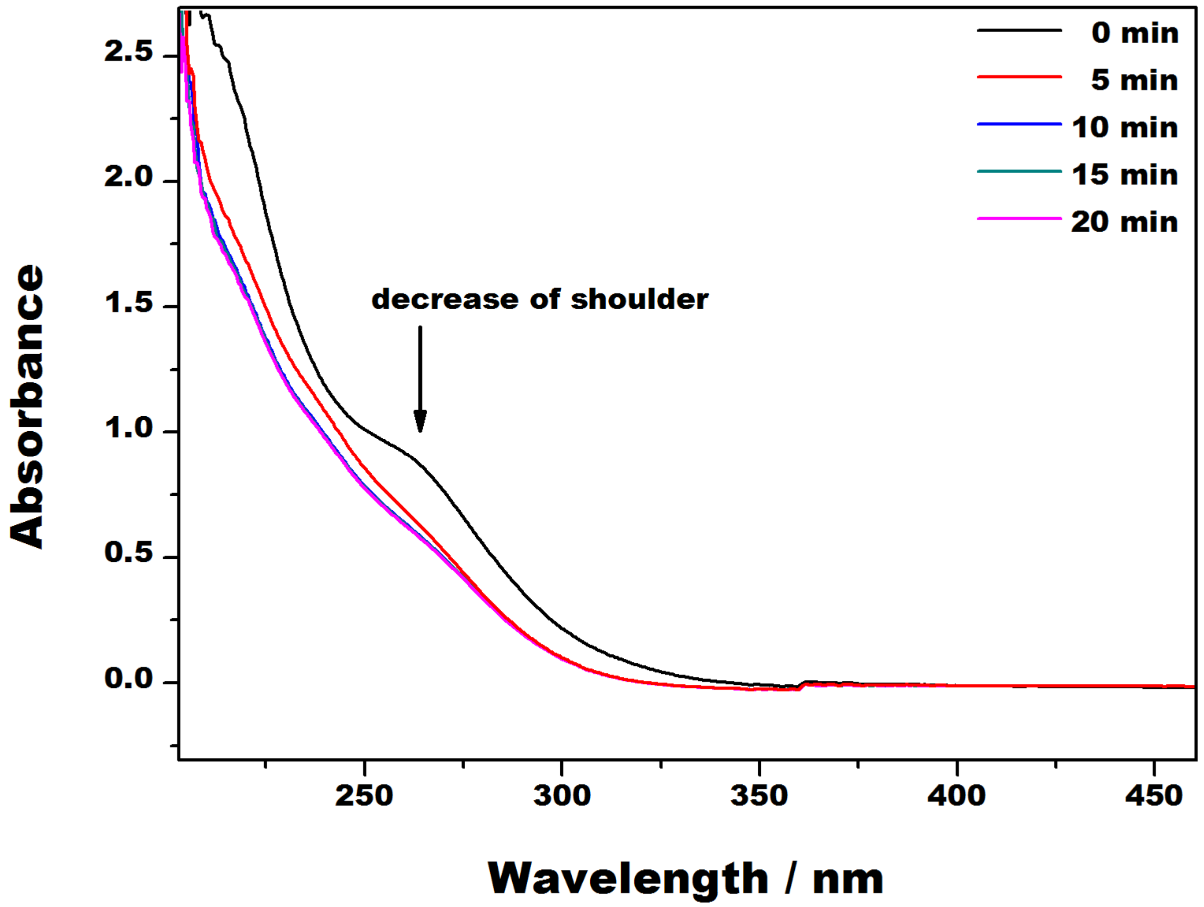

Figure S20. UV-vis spectra of **Co_{20}** (8.4 μM) in unbuffered aqueous solution (pH = 0.7 via HCl [2 M]) are characterized by a stepwise decrease of the shoulder at ~250 nm indicating degradation of the POT framework.

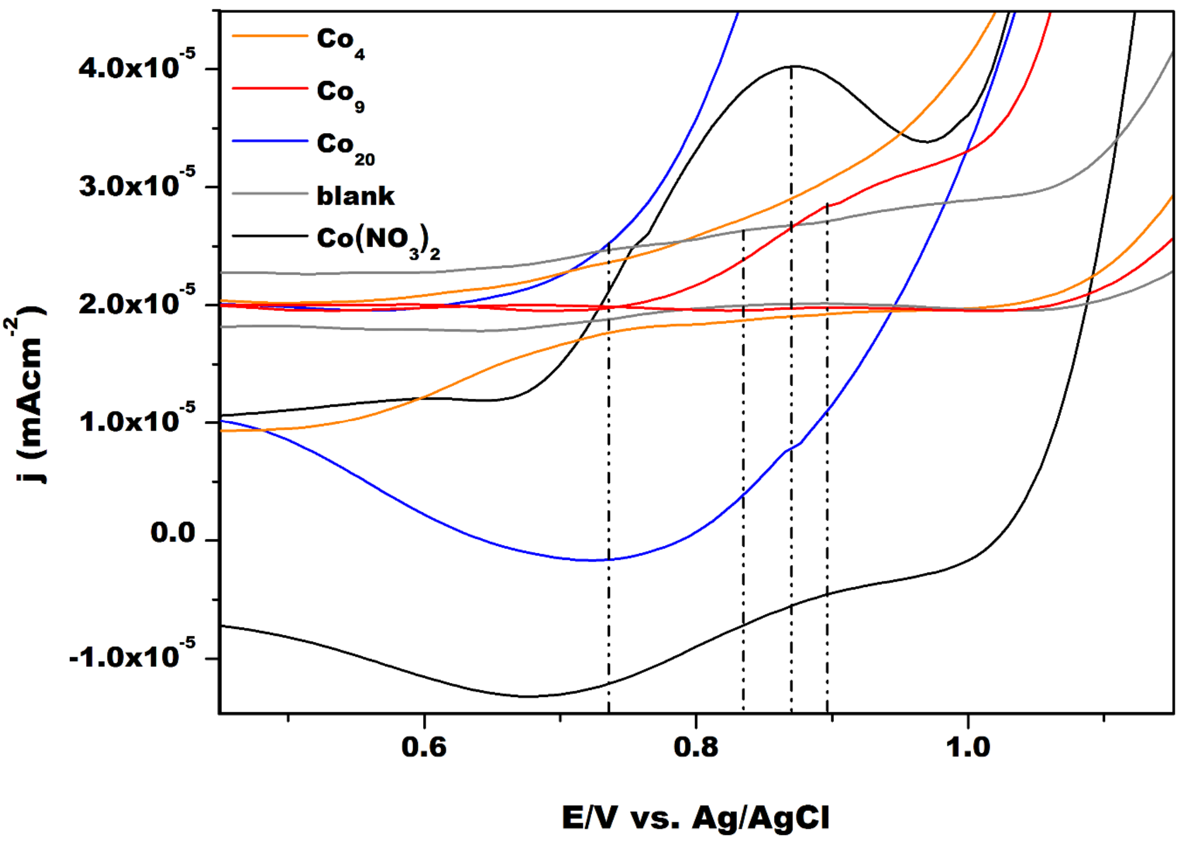

Figure S21. Superimposed cyclic voltammograms of **Co_4**, **Co_9**, **Co_{20}**, $Co(NO_3)_2$ and buffer-only solution at a 100 mV s^{-1} scan rate with the highlighted potential range where Co^{2+} gets oxidized to Co^{3+}.

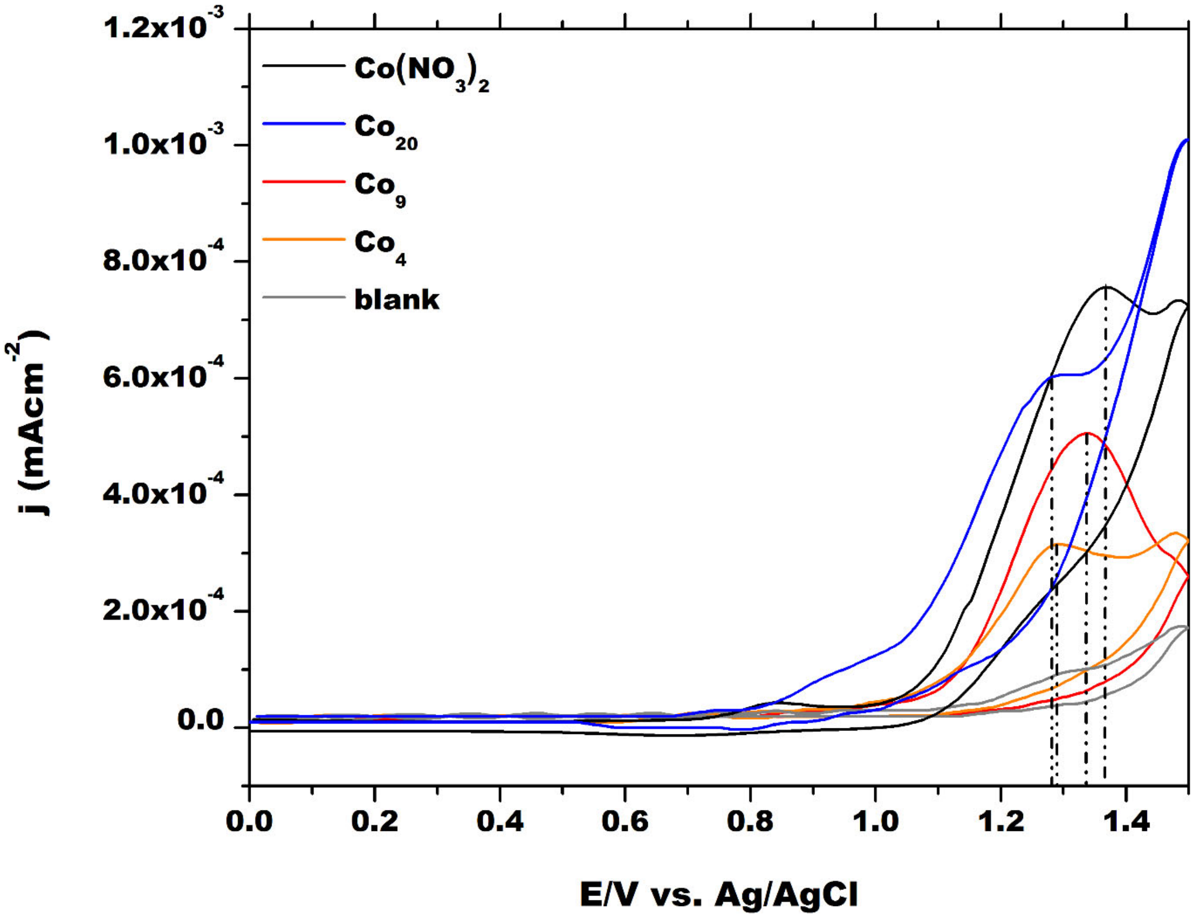

Figure S22. Superimposed cyclic voltammograms of **Co_4**, **Co_9**, **Co_{20}**, $Co(NO_3)_2$ and buffer-only solution at a 100 mV s^{-1} scan rate.

Table S12. Anodic potentials (E_{pa}) and currents (i_{pa}) of peaks obtained by cyclic voltammetry from **Co_4**, **Co_9**, **Co_{20}**, and $Co(NO_3)_2$ at pH = 8 in 80mM sodium borate buffer.

Cobalt-containing compound	v / (mV^{-1})	E_{pa}/V	i_{pa}/($mAcm^{-2}$)	attribution
**Co_4**	100	0.84	2.79 x 10^{-5}	Co^{2+} -> Co^{3+}
		1.29	3.12 x 10^{-4}	water oxidation
**Co_9**	100	0.89	2.892.79 x 10^{-5}	Co^{2+} -> Co^{3+}
		1.34	5.01 x 10^{-4}	water oxidation
**Co_{20}**	100	0.74	2.782.79 x 10^{-5}	Co^{2+} -> Co^{3+}
		1.28	6.03 x 10^{-4}	water oxidation
$Co(NO_3)_2$	100	4.29	0.842.79 x 10^{-5}	Co^{2+} -> Co^{3+}
		1.37	7.55 x 10^{-4}	water oxidation

10. Visible light-driven water oxidation

10.1. Impact of POM-WOC (**Co₄**, **Co₉** and **Co₂₀**) concentrations

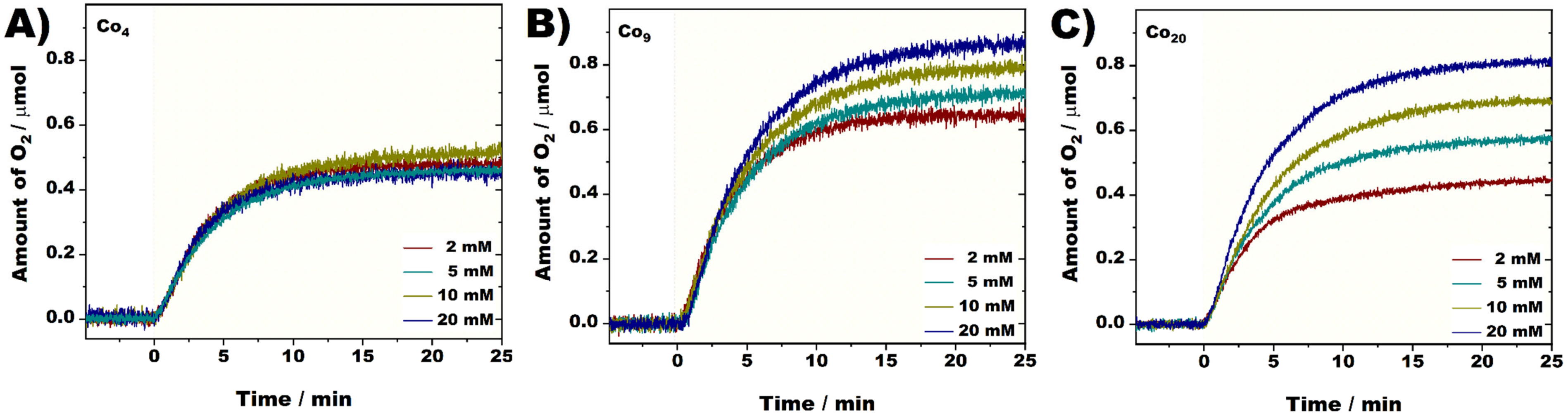

Figure S23. As-recorded O_2 evolution profiles for **A) Co_4**, **B) Co_9** and **C) Co_{20}**, measured for 2, 5, 10 and 20 µM POM concentration values in POM solutions buffered in 80 mM borate buffer at pH 8 and containing $Na_2S_2O_8$ (5 mM) and $[Ru(bpy)_3]^{2+}$ (1 mM) as an oxidant and a photosensitizer, respectively.

10.2. Impact of $Na_2S_2O_8$ and $[Ru(bpy)_3]^{2+}$ concentrations

For all the WOC experiments performed under standard conditions, the O_2 evolution reaches a plateau after illumination for some time (**Figure 2A**, **Figure S23**). Besides, the activity-concentration trends (**Figure 2B**) reveal that other experimental parameters could govern the activity under the experimental conditions set. To elucidate these factors, additional tests were performed and indeed a pronounced impact of [SA] and [PS] on the WOC performance was found. **Figure S24** illustrates that as much as 445 % of the initial O_2 amount was recorded when $[Ru(bpy)_3]^{2+}$ concentration was increased by 5 times (**Figure S24A**), and a close to linear increase was also recorded when double the concentration of the $Na_2S_2O_8$ was used (**Figure S24B**).

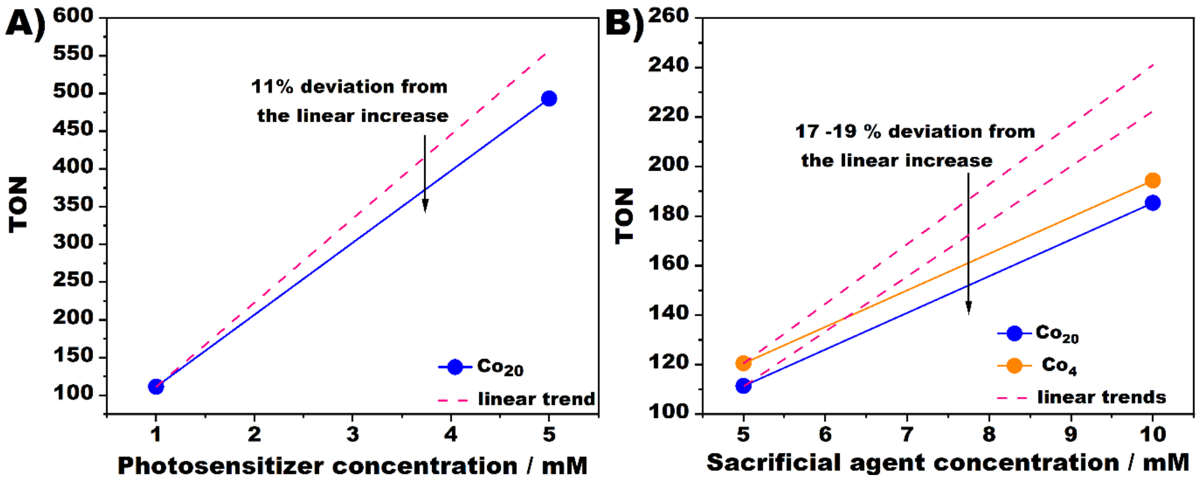

Figure S24. Amount of oxygen evolved (expressed in TONs) for 2 µM POM solutions buffered in 80 mM borate buffer at pH 8 and containing 5 mM $Na_2S_2O_8$ and 1 mM $[Ru(bpy)_3]^{2+}$ as a standard (points on the left bottom of the graphs) **A)** for **Co_{20}** when $[Ru(bpy)_3]^{2+}$ concentration was increased from 1 to 5 mM and **B)** for **Co_4** and **Co_{20}** when $Na_2S_2O_8$ was increased from 5 to 10 mM.

11. POM WOC activity and integrity

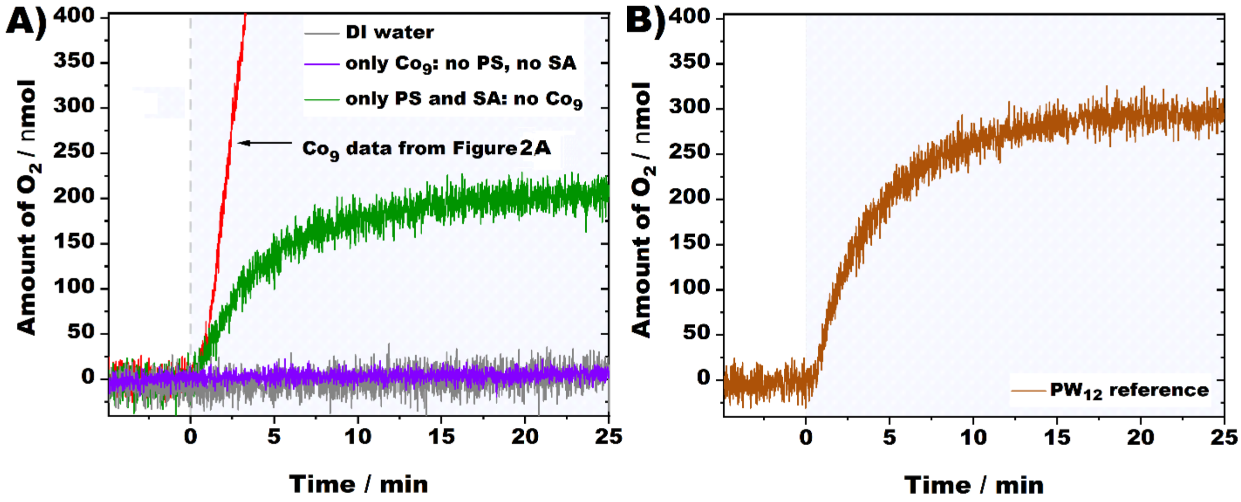

Figure S25. As-recorded O_2 evolution profiles for **A)** blank experiments using (i) pure DI water, (ii) only 20 μM **Co_9** containing solution and (iii) the 80 mM borate buffer (pH 8) containing $Na_2S_2O_8$ (5 mM) and $[Ru(bpy)_3]^{2+}$ (1 mM) without any POM WOC. **B)** reference experiments using 20 μM **$[PW_{12}O_{40}]^{3-}$ {PW_{12}}** solution in the 80 mM borate buffer (pH 8) containing $Na_2S_2O_8$ (5 mM) and $[Ru(bpy)_3]^{2+}$ (1 mM).

11.1 Co-GT recyclability

To investigate the recyclability of **Co₉**, the reaction mixture containing **Co₉** (20 µM) along with $[Ru(bpy)_3]Cl_2$ (1 mM) and $Na_2S_2O_8$ (5 mM) in 80 mM borate buffer at pH 8 (V_{total} = 2 mL) was reloaded with similar amounts of $[Ru(bpy)_3]Cl_2$ and $Na_2S_2O_8$ after saturation in WOC activity indicated the end of the first illumination cycle. Illumination of the reaction mixture in a second reaction cycle resulted in observable WOC activity (**Figure S26**) thereby demonstrating the recyclability of the Co-GT catalyst.

In a consecutive experiment involving **Co₉** (20 µM) along with $[Ru(bpy)_3]Cl_2$ (1 mM) and $Na_2S_2O_8$ (5 mM) in 80 mM borate buffer at pH 8, **Co₉** was removed from the system upon extraction using Tetraheptylammonium nitrate ($THpANO_3$) [33] after the first illumination cycle was finished. Reloading of the remaining reaction mixture with $[Ru(bpy)_3]Cl_2$ (1 mM) and $Na_2S_2O_8$ (5 mM) and subsequent illumination to trigger a second reaction cycle showed WOC activity resembling that of a blank test (**Figure S26**).

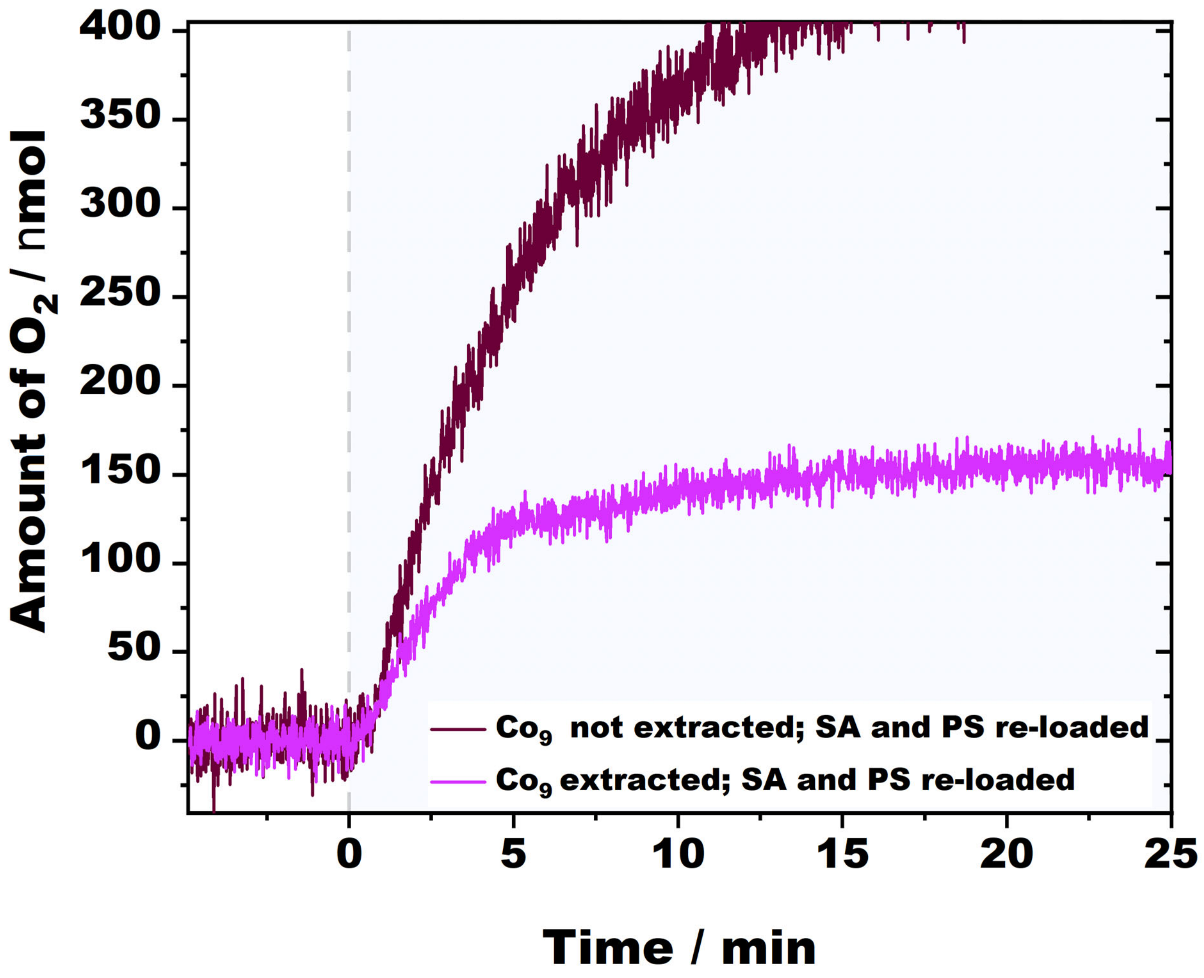

Figure S26 POM extraction experiments: (i) second illumination cycle was performed after **Co₉** POM was extracted from the reaction solution that was re-loaded with $Na_2S_2O_8$ (5 mM) and $[Ru(bpy)_3]^{2+}$ (1 mM) and (ii) second illumination cycle was performed without **Co₉** POM extraction from the reaction solution, but the solution was still re-loaded with $Na_2S_2O_8$ (5 mM) and $[Ru(bpy)_3]^{2+}$ (1 mM). When Co-GT was extracted from the reaction solution before the photocatalytic reaction was started, the level of O_2 resembled that of a blank test lacking the polyanion, which confirms the efficiency of the extraction procedure and highlights the catalytic activity of **Co₉**.

11.2 X-ray fluorescence analysis: Extraction procedure of **Co_4, Co_9 and Co_{20}** for X-ray fluorescence analysis

2 ml of sodium borate buffer (80 mM, pH = 8.0; 1 mM $[Ru(bpy)_3]Cl_2$; 5 mM $Na_2S_2O_8$) containing **Co_4** (20 µM), **Co_9** (20 µM) or **Co_{20}** (20 µM) was irradiated for 30 min. Then **Co_4**, **Co_9** and **Co_{20}** were extracted with tetraheptylammoniumnitrate, which was synthesized according to a published procedure [33], out of the aqueous phase into toluene as described in the previous section. The aqueous phase was collected and analyzed with X-Ray fluorescence spectroscopy. The final concentration of the analyzed POMs was 0.2 µM.

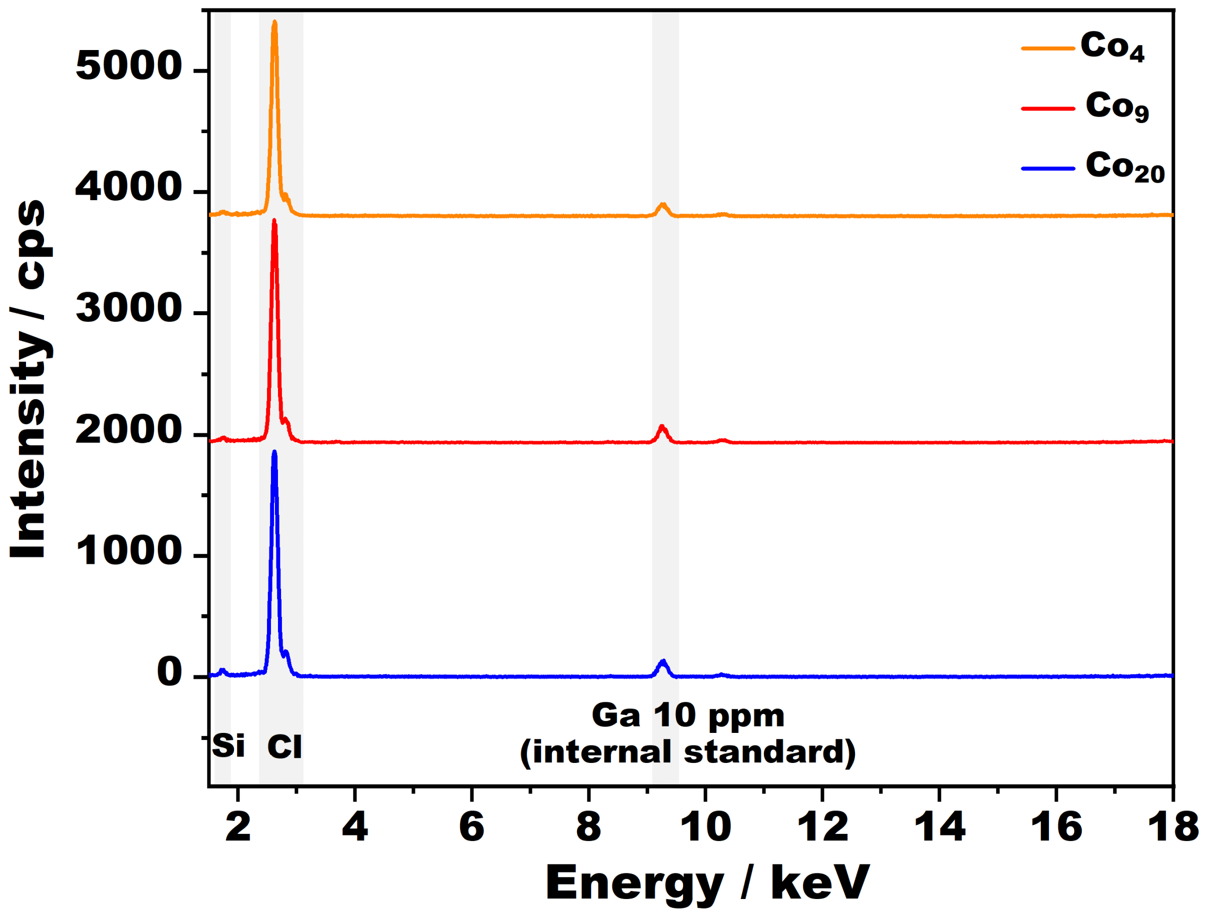

Figure S27. X-ray fluorescence spectra (intensity in counts per seconds = cps *vs* energy in keV) of **Co_4**, **Co_9** and **Co_{20}**. The XRF spectra of the three aqueous phases look similar with only one significant peak corresponding to Cl^- originating from $[Ru(bpy)_3]Cl_2$ whereas $[Ru(bpy)_3]^{2+}$ has been transferred to the organic phase along with the corresponding Co-GT (**Co_4**, **Co_9** or **Co_{20}**, respectively) and hence is not observed in the spectra. The other peaks seen are Ga with a known concentration used as the internal standard for quantification, and Si as the samples are mounted on reflectors made of Si.

Table S13. Presentation of the ppm of elements present in 5 ml of WOC reaction medium after extraction of **Co_9** into organic phase using $THANO_3$.

POM	Element		Weight %	ppm
**Co_4**	P;	Kα;	8.51E-01	30.42
	S;	Kα;	2.81E+00	100.39
	Cl;	Kα;	9.60E+01	3434.95
	Co;	Kα;	2.95E-03	0.11
	Ga;	Kα;	2.80E-01	10
	Ge;	Kα;	1.10E-03	0.04
	W;	Lα;	1.02E-02	0.36
**Co_9**	P;	Kα;	1.38E+00	44.5
	S;	Kα;	2.23E+00	72.03
	Cl;	Kα;	9.61E+01	3102.37
	Ga;	Kα;	3.10E-01	10
	W;	Lα;	1.10E-02	0.36
**Co_{20}**	Na;	Kα;	7.05E+00	258.31
	P;	Kα;	6.03E-01	22.09
	S;	Kα;	2.14E+00	78.44
	Cl;	Kα;	8.99E+01	3292.41
	Co;	Kα;	2.42E-03	0.09
	Ga;	Kα;	2.73E-01	10
	W;	Lα;	1.67E-02	0.61

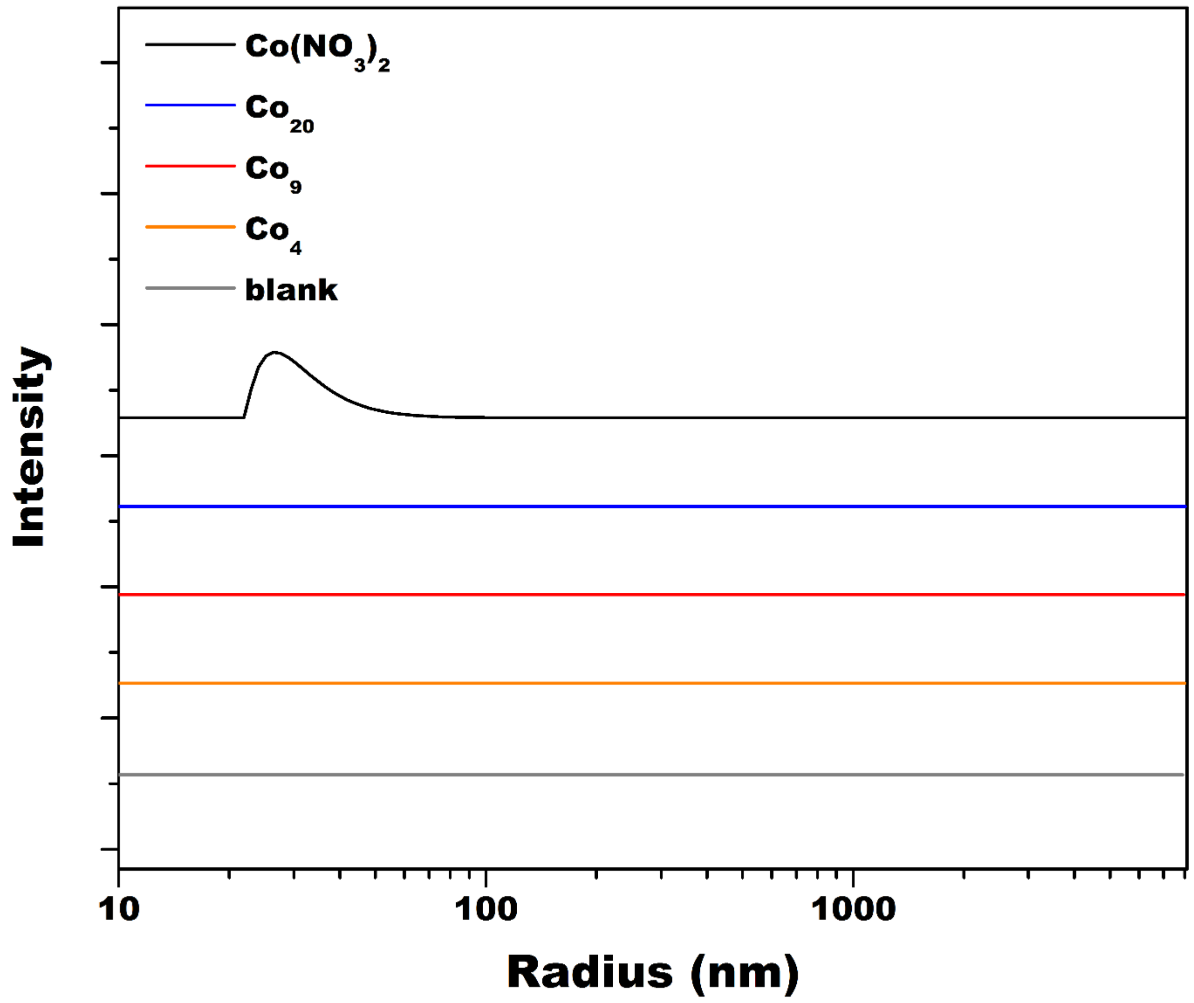

Figure S28. DLS curves of the post-catalytic water oxidation reaction solutions containing **Co_4**, **Co_9**, **Co_{20}** or $Co(NO_3)_2$ (20 μM), $[Ru(bpy)_3]^{2+}$ (1 mM) and $Na_2S_2O_8$ (5 mM) in an 80 mM borate buffer solution (pH 8) after 30 min of irradiation. In contrast to the blank lacking any catalyst and the Co-GT (**Co_4**, **Co_9**, **Co_{20}**) containing solutions, the DLS curve of the irradiated reaction mixture with $Co(NO_3)_2$ displays a peak at ~26.4 nm indicating the formation of cobalt nanoparticles.

11.3 Post-catalytic POM-precipitation for subsequent analysis with FTIR

The photocatalytic reaction was carried out with 200 µM (**Co_4** and **Co_{20}**) and 400 µM (**Co_9**) of the corresponding catalyst, $[Ru(bpy)_3]Cl_2$ (1 mM), and $Na_2S_2O_8$ (5 mM) in 2 ml of sodium borate buffer (80 mM, pH = 8.0) to ensure sufficient amounts for post-analysis. After 30 min of illumination solid cesium chloride was added to the reaction mixture resulting in the immediate formation of precipitates. The precipitate was centrifuged at 2500 rpm for 5 min and completeness of the precipitation was insured by adding cesium chloride to the supernatant. The precipitates were air dried and displayed to IR-spectroscopic analysis.

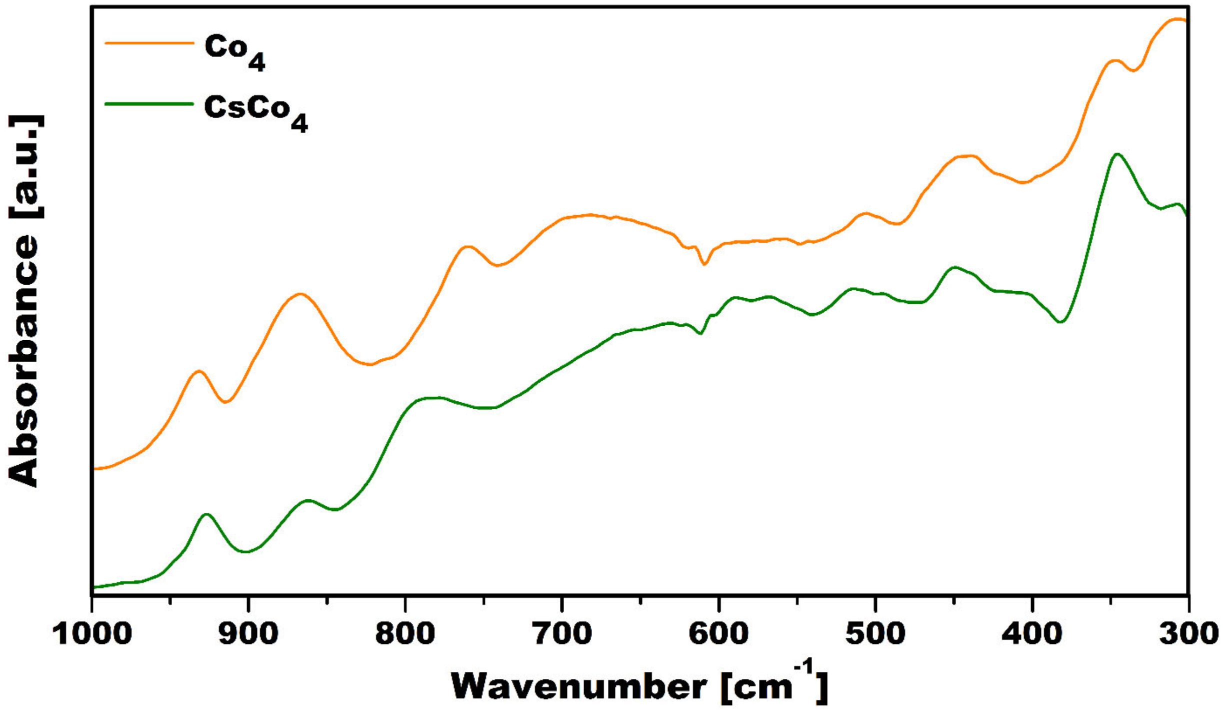

Figure S29. FTIR spectra showing the tungsten fingerprint area of **Co_4** before (orange) and after photocatalysis and precipitation as cesium salt **$CsCo_4$** (green).

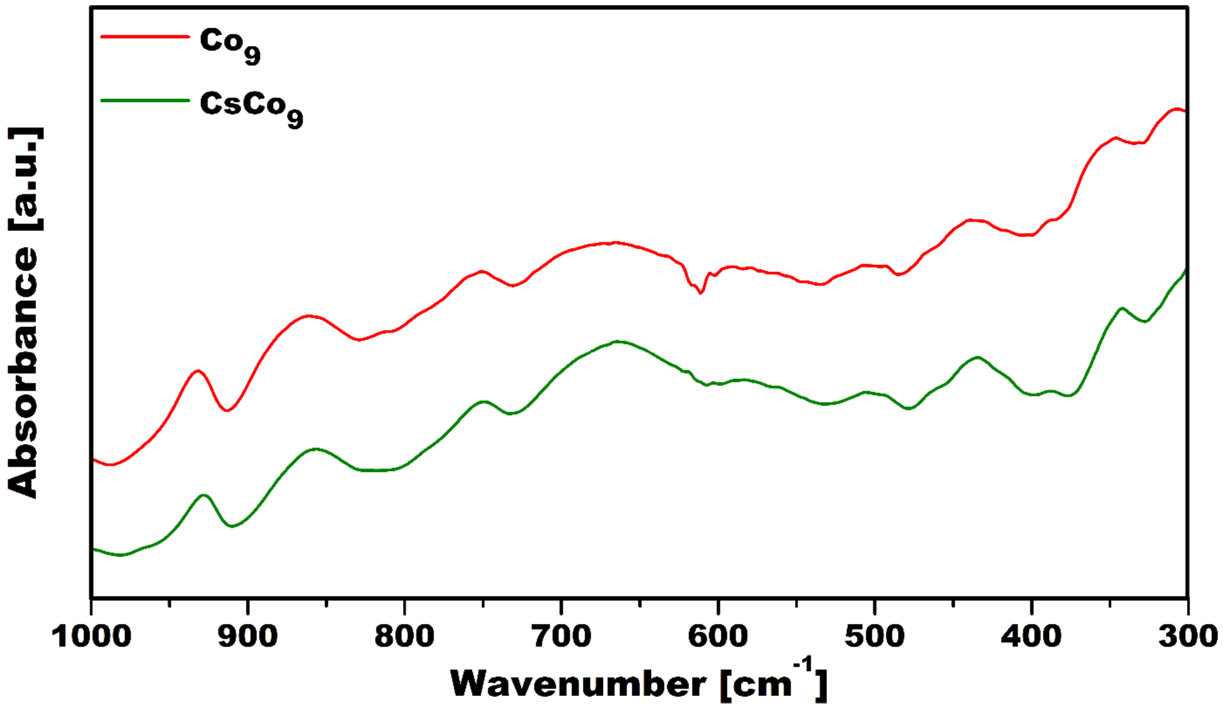

Figure S30. FTIR spectra showing the tungsten fingerprint area of **Co_9** before (red) and after photocatalysis and precipitation as cesium salt **$CsCo_9$** (green).

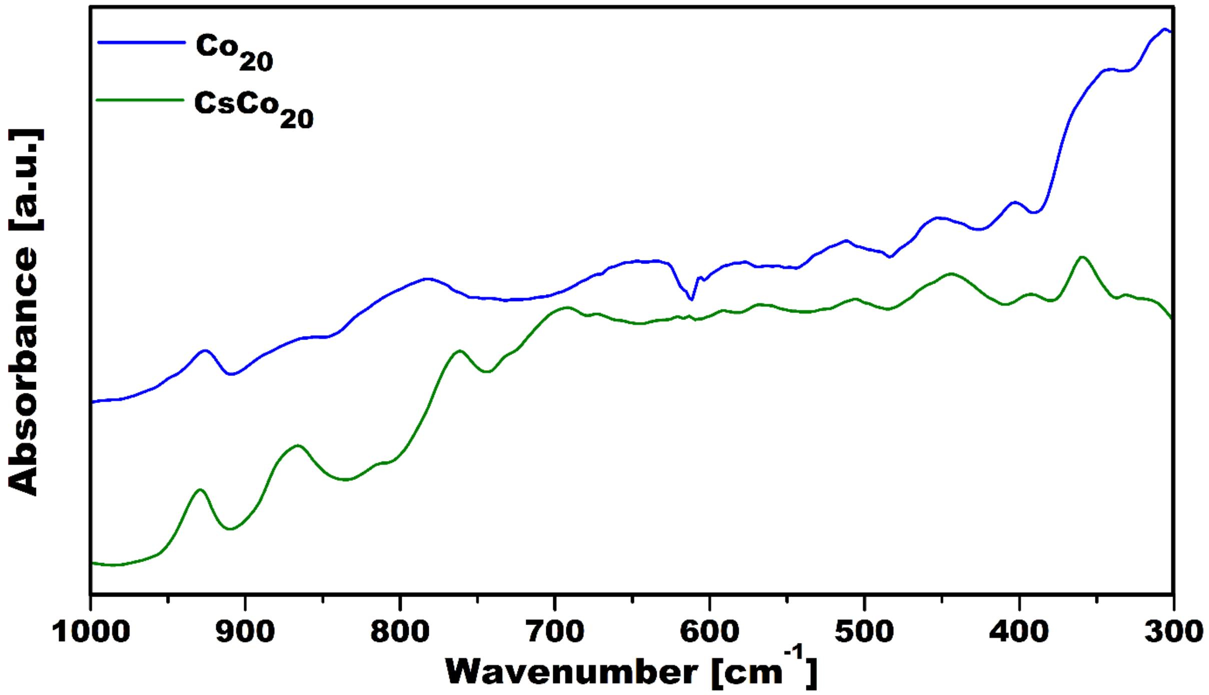

Figure S31. FTIR spectra showing the tungsten fingerprint area of **Co_{20}** before (blue) and after photocatalysis and precipitation as cesium salt **$CsCo_{20}$** (green).

12. Photoluminescence (PL) emission spectroscopy

Based on the Stern-Volmer analyses, the rate constant for oxidative quenching by $Na_2S_2O_8$ is deduced to be $8.48*10^8$ $M^{-1}s^{-1}$, and for the reductive quenching by **Co_9**, it is found out to be $38.18*10^9$ $M^{-1}s^{-1}$. However, the linear fit of the **Co_9** dataset only yields the R^2 of 0.927. If only first four data points are fitted (yielding R^2 of 0.997), a rate constant of $57.93*10^9$ $M^{-1}s^{-1}$ can be deduced. In this case, the deviation of the 20 µM data point from this trend can be a result of intermolecular quenching due to increased POM-POM interactions, which cannot be excluded at higher POM concentrations, but would not change the main qualitative conclusion.

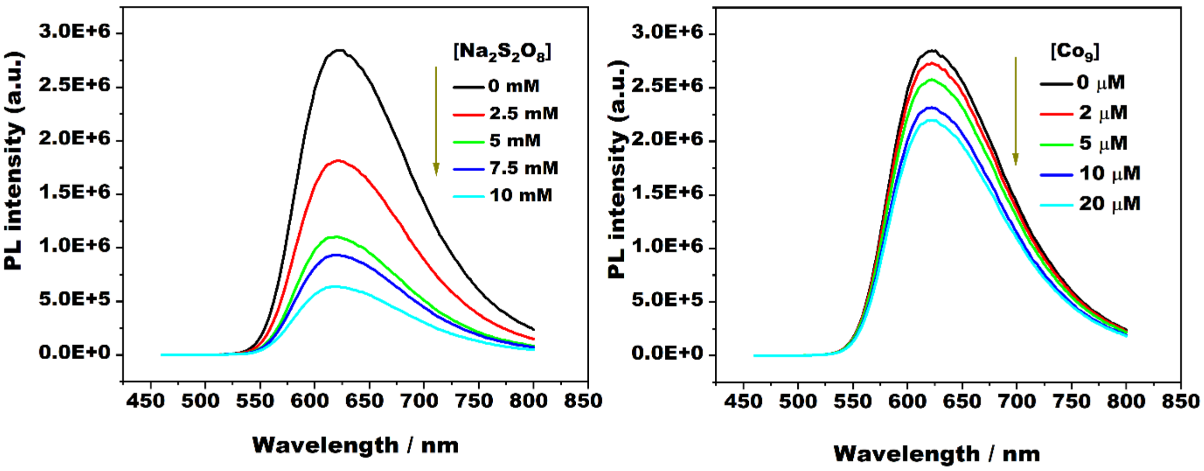

Figure S32. PL emission quenching of $[Ru(bpy)_3]Cl_2$ (1 mM) excited at 445 nm by addition of different amounts of (a) $Na_2S_2O_8$ and (b) **Co_9** catalyst [From linear fitting of Stern-Volmer plots, the calculated K_q values for the spectra were (a) $8.48*10^8$ $M^{-1}s^{-1}$ and (b) $38.18*10^9$ $M^{-1}s^{-1}$ respectively].

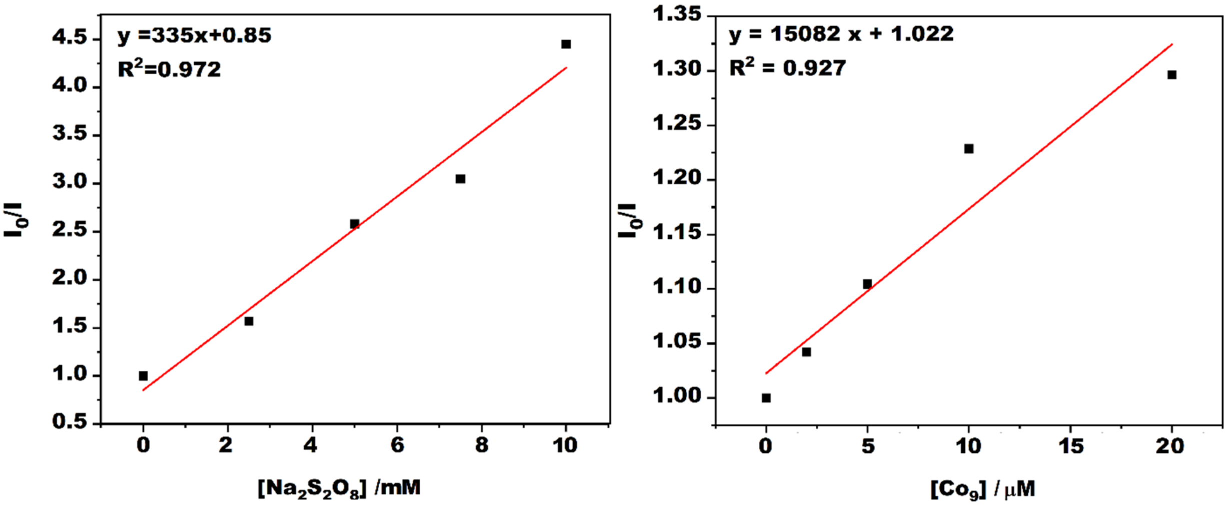

Figure S33. Stern-Volmer plots of emission quenching (where I_0 and I are the fluorescence intensities in the absence and presence of the quencher) of $[Ru(bpy)_3]Cl_2$ solution (20 µM) by (a) $Na_2S_2O_8$ and (b) **Co_9** catalyst.

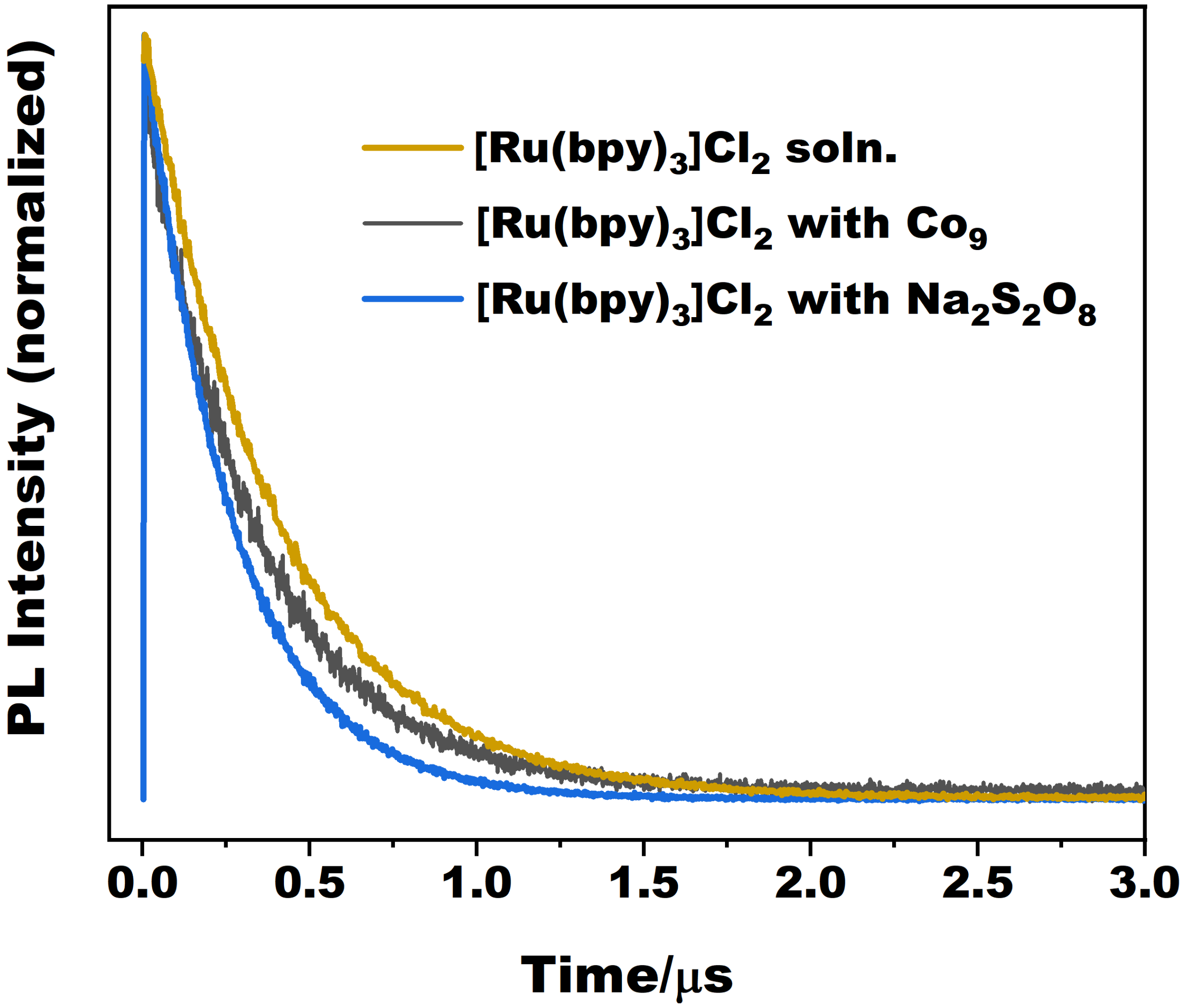

Figure S34. PL time-resolved spectra of $[Ru(bpy)_3]Cl_2$ (1 mM) solution, $[Ru(bpy)_3]Cl_2$ with $Na_2S_2O_8$ (10 mM), and $[Ru(bpy)_3]Cl_2$ with $\mathbf{Co_9}$ (20 μM), detected at 620 nm, excited with a laser wavelength 377 nm.

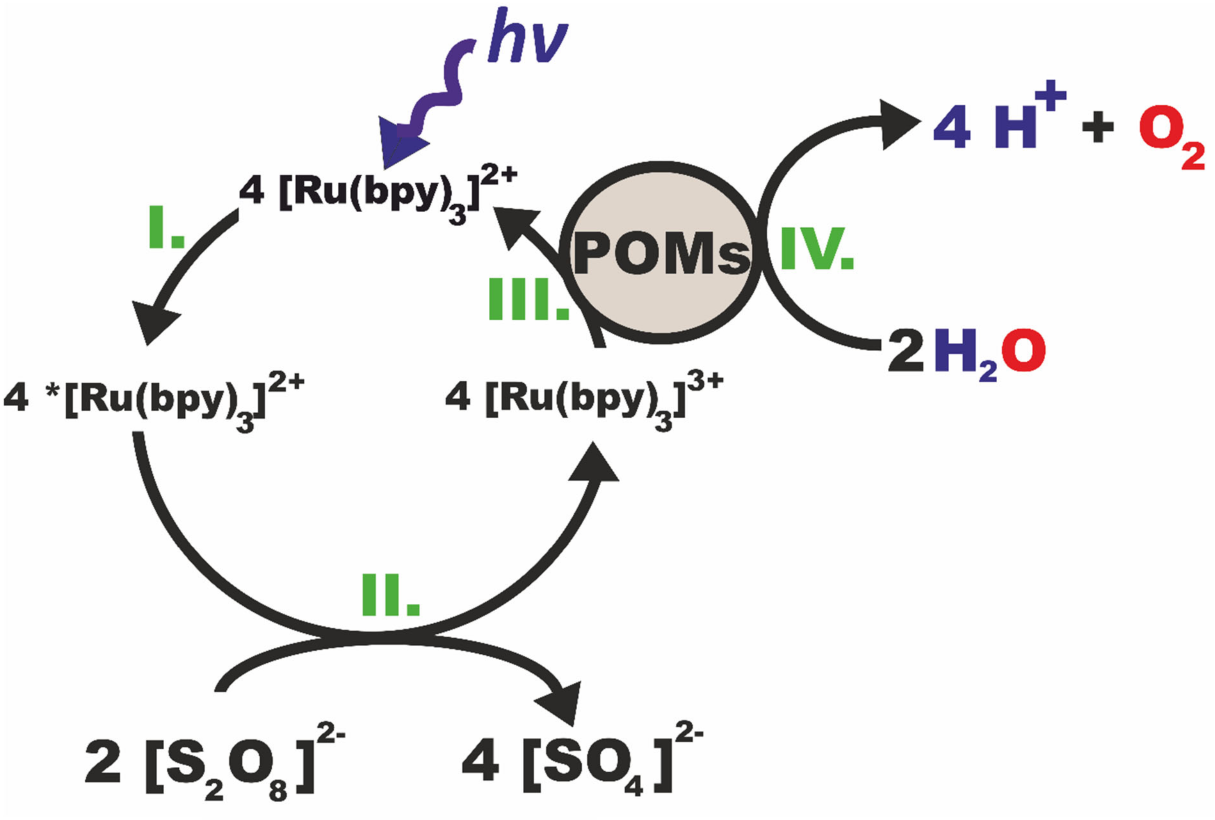

Scheme S1. Photocatalytic oxidative quenching mechanism suggested based on the photoluminescence experiments. The reaction starts with the irradiation of the photosensitizer $[Ru(bpy)_3]^{2+}$ (step I.) thereby creating a $^*[Ru(bpy)_3]^{2+}$ triplet excited state. In a second step (step II.), the $^*[Ru(bpy)_3]^{2+}$ reduces the sacrificial agent $[S_2O_8]^{2-}$ to SO_4^{2-} upon transferring a total of 4 electrons and creating $[Ru(bpy)_3]^{3+}$ (step III.). Reduction of the $[Ru(bpy)_3]^{3+}$ to the initial $[Ru(bpy)_3]^{2+}$ takes place upon donation of 4 electrons from the corresponding POM. The oxidized POM is reduced to its initial state by oxidizing two equivalents of H_2O to molecular O_2 in a 4 – electron process (step IV.).

13. References

[1] a) L.-H. Bi, U. Kortz, S. Nellutla, A. C. Stowe, J. van Tol, N. S. Dalal, B. Keita, L. Nadjo, Structure, Electrochemistry, and Magnetism of the Iron(III)-Substituted Keggin Dimer, $[Fe_6(OH)_3(A\text{-}\alpha\text{-}GeW_9O_{34}(OH)_3)_2]^{11-}$. *Inorg. Chem.* **2005**, *44*, 896-903; b) N. H. Nsouli, B. S. Bassil, M. H. Dickman, U. Kortz, B. Keita, L. Nadjo, Synthesis and Structure of Dilacunary Decatungstogermanate, $[\gamma\text{-}GeW_{10}O_{36}]^{8-}$. *Inorg. Chem.* **2006**, *45*, 3858-3860; c) Z. Huang, Z. Luo, Y. V. Geletii, J. W. Vickers, Q. Yin, D. Wu, Y. Hou, Y. Ding, J. Song, D. G. Musaev, C. L. Hill, T. Lian, Efficient Light-Driven Carbon-Free Cobalt-Based Molecular Catalyst for Water Oxidation. *J. Am. Chem. Soc.* **2011**, *133*, 2068-2071; d) Q. Yin, J. M. Tan, C. Besson, Y. V. Geletii, D. G. Musaev, A. E. Kuznetsov, Z. Luo, K. I. Hardcastle, C. L. Hill, A Fast Soluble Carbon-Free Molecular Water Oxidation Catalyst Based on Abundant Metals. *Science*, **2010**, *328*, 342-345; e) M. Phillips, The preparation of phosphotungstic acid and of sodium and barium phosphotungstates. *J. Soc. Chem. Ind.*, **1950**, *69*, 282-284.

[2] Bruker SAINT v7.68A Copyright © **2005-2016** Bruker AXS

[3] G. M. Sheldrick, University of Göttingen, Germany **1996**.

[4] G. M. Sheldrick, *Acta Cryst.* A64 **2008**, 112-122.

[5] O. V. Dolomanov, L. J. Bourhis, R. J. Gildea, J. A. K. Howard, H. Puschmann, *OLEX2*: a complete structure solution, refinement and analysis program. *J. Appl. Cryst.* **2009**, *42*, 339-341.

[6] C. B. Hübschle, G. M. Sheldrick, B. Dittrich, *ShelXle*: a Qt graphical user interface for *SHELXL*. *J. Appl. Cryst.* **2011**, *44*, 1281-1284.

[7] L. Chen, D. Shi, J. Zhao, Y. Wang, P. Ma, J. Wang, J. Niu, Three Transition-Metal Substituted Polyoxotungstates Containing Keggin Fragments: From Trimer to One-Dimensional Chain to Two-Dimensional Sheet. *Cryst. Growth Des.* **2011**, *11*, 1913-1923.

[8] M. Ibrahim, V. Mereacre, N. Leblanc, W. Wernsdorfer, C. E. Anson, A. K. Powell, Self-Assembly of a Giant Tetrahedral 3 d–4 f Single-Molecule Magnet within a Polyoxometalate System. *Angew. Chem. Int. Ed.* **2015**, *54*, 15574-15578; *Angew. Chem.* **2015**, *127*, 15795-15799.

[9] T. Ruizhan, L. Chen, Y. Liu, B. Liu, G. Xue, H. Hu, F. Fu, J. Wang, A new multi-cobalt-substituted C-shaped polyoxotungstogermanate, $[((CoOH_2)Co_2GeW_9O_{34})_2(GeW_6O_{26})]^{20-}$ *Inorg. Chem. Commun.* **2010**, *13*, 98-100.

[10] J. Wang, P. Ma, Y. Shen, J. Niu, Tetra-Transition-Metal Substituted Weakley-Type Sandwich Germanotungstates and their Derivatives Decorated by Transition-Metal Complexes. *Cryst. Growth Des.* **2008**, *8*, 3130-3133.

[11] Z. Zhang, E. Wang, Y. Li, H. An, Y. Qi, L. Xu, Synthesis, characterization, and crystal structures of two 6-cobalt-containing dimeric polyoxoanions: $[Co_2(H_2O)_{10}Co_4(H_2O)_2(B\text{-}\alpha\text{-}XW_9O_{34})_2]^{8-}$ (X = Ge and Si). *J. Mol. Struct.* **2008**, *872*, 176-181.

[12] C.-L. Wang, S.-X. Liu, C.-Y. Sun, L.-H. Xie, Y.-H. Ren, D.-D. Liang, H.-Y. Cheng, Bimetals substituted germanotungstate complexes with open Wells-Dawson structure: Synthesis, structure, and electrochemical behavior of $[\{M(H_2O)\}(\mu\text{-}H_2O)_2K\{M(H_2O)_4\}(Ge_2W_{18}O_{66})]^{11-}$ (M = Co, Ni, Mn). *J. Mol. Struct.* **2007**, *841*, 88-95.

[13] N. H. Nsouli, A. H. Ismail, I. S. Helgadottir, M. H. Dickman, J. M. Clemente-Juan, U. Kortz, Copper-, Cobalt-, and Manganese-Containing 17-Tungsto-2-Germanates. *Inorg. Chem.* **2009**, *48*, 5884-5890.

[14] X.-B. Han, Z.-M. Zhang, T. Zhang, Y.-G. Li, W. Lin, W. You, Z.-M. Su, E.-B. Wang, Polyoxometalate-Based Cobalt–Phosphate Molecular Catalysts for Visible Light-Driven Water Oxidation. *J. Am. Chem. Soc.* **2014**, *136*, 5359-5366.

[15] C.-Y. Sun, S.-X. Liu, C.-L. Wang, L.-H. Xie, C.-D. Zhang, B. Gao, Z.-M. Su, H.-Q. Jia, Synthesis, structure and characterization of a new cobalt-containing germanotungstate with open Wells-Dawson structure: $K_{13}[\{Co(H_2O)\}(\mu\text{-}H_2O)_2K(Ge_2W_{18}O_{66})]$. *J. Mol. Struct.* **2006**, *785*, 170-175.

[16] L. Chen, D. Shi, J. Zhao, Y. Wang, P. Ma, J. Niu, Synthesis, structure and magnetism of a 2-D organic–inorganic hybrid tetra-CoII-substituted sandwich-type Keggin germanotungstate: $\{[Co(dap)_2(H_2O)]_2[Co(dap)_2]_2[Co_4(Hdap)_2(B\text{-}\alpha\text{-}HGeW_9O_{34})_2]\}\cdot 7H_2O$ *Inorg. Chem. Commun.* **2011**, *14*, 1052-1056.

[17] K. Dong, P. Ma, H. Wu, Y. Wu, J. Niu, J. Wang, Cobalt- and Nickel-Containing Germanotungstates Based on Open Wells–Dawson Structure: Synthesis and Characterization of Tetrameric Anion. *Inorg. Chem.* **2019**, *58*, 6000-6007.

[18] J. Wang, J. Liu, J. Niu, Hydrothermal synthesis and structural characterization of two inorganic–organic composite sandwich-type tungstogermanates. *J. Coord. Chem.* **2009**, *62*, 3599-3605.

[19] S. Cao, L. Piao, Considerations for a More Accurate Evaluation Method for Photocatalytic Water Splitting. *Angew. Chem. Int. Ed.* **2020**, *59*, 18312-18320. *Angew. Chem.* **2020**, *132*, 18468-18476.

[20] M. Natali, I. Bazzan, S. Goberna-Ferrón, R. Al-Oweini, M. Ibrahim, B. Bassil, H. Dau, F. Scandola, J. Galán-Mascarós, U. Kortz, Photo-assisted water oxidation by high-nuclearity cobalt-oxo cores: tracing the catalyst fate during oxygen evolution turnover. *Green Chem.* **2017**, *19*, 2416-2426.

[21] X.-B. Han, Z.-M. Zhang, T. Zhang, Y.-G. Li, W. Lin, W. You, Z.-M. Su, E.-B. Wang, Polyoxometalate-Based Cobalt–Phosphate Molecular Catalysts for Visible Light-Driven Water Oxidation. *J. Am. Chem. Soc.* **2014**, *136*, 5359-5366.

[22] H. Lv, J. Song, Y. V. Geletii, J. W. Vickers, J. M. Sumliner, D. G. Musaev, P. Kögerler, P. F. Zhuk, J. Bacsa, G. Zhu, An Exceptionally Fast Homogeneous Carbon-Free Cobalt-Based Water Oxidation Catalyst. *J. Am. Chem. Soc.* **2014**, *136*, 9268-9271.

[23] G. Zhu, Y. V. Geletii, P. Kögerler, H. Schilder, J. Song, S. Lense, C. Zhao, K. I. Hardcastle, D. G. Musaev, C. L. Hill, Water oxidation catalyzed by a new tetracobalt-substituted polyoxometalate complex: $[\{Co_4(\mu\text{-}OH)(H_2O)_3\}(Si_2W_{19}O_{70})]^{11-}$. *Dalton Trans.* **2012**, *41*, 2084-2090.

[24] Y. Zhang, Y. Wang, X. Meng, L. Yu, Y. Ding, M. Chen, Binuclear polyoxometalates based on abundant metals as efficient homogeneous photocatalytic water oxidation catalysts. *J. Photochem. Photobiol. A* **2018**, *355*, 371-376.

[25] W.-C. Chen, X.-L. Wang, C. Qin, K.-Z. Shao, Z.-M. Su, E.-B. Wang, A carbon-free polyoxometalate molecular catalyst with a cobalt–arsenic core for visible light-driven water oxidation. *Chem. Commun.* **2016**, *52*, 9514-9517.

[26] R. Xiang, Y. Ding, J. Zhao, Visible-Light-Induced Water Oxidation Mediated by a Mononuclear-Cobalt(II)-Substituted Silicotungstate. *Chem. Asian J.* **2014**, *9*, 3228-3237.

[27] J. Wei, Y. Feng, P. Zhou, Y. Liu, J. Xu, R. Xiang, Y. Ding, C. Zhao, L. Fan, C. Hu, A Bioinspired Molecular Polyoxometalate Catalyst with Two Cobalt(II) Oxide Cores for Photocatalytic Water Oxidation. *ChemSusChem* **2015**, *8*, 2630-2634.

[28] L. Gong, L. Yu, K. Yu, Y. Ding, J. Lv, C. Wang, Z. Su, B. Zhou, Efficient visible light-driven water oxidation catalysts based on *B*-β-$\{BiW_8O_{30}\}$ and unique 14-nuclear hetero-metal sandwich unit. *Chem. Commun.* **2018**, *54*, 674-677.

[29] F. Song, Y. Ding, B. Ma, C. Wang, Q. Wang, X. Du, S. Fu, J. Song, $K_7[Co^{III}Co^{II}(H_2O)W_{11}O_{39}]$: a molecular mixed-valence Keggin polyoxometalate catalyst of high stability and efficiency for visible light-driven water oxidation. *Energy Environ. Sci.* **2013**, *6*, 1170-1184.

[30] a) Q.-G. Zhai, X.-Y. Wu, S.-M. Chen, Z.-G. Zhao, C.-Z. Lu. Construction of Ag/1,2,4-Triazole/Polyoxometalates Hybrid Family Varying from Diverse Supramolecular Assemblies to 3-D Rod-Packing Framework. *Inorg. Chem.* **2007**, *46*, 5046–5058; b) J.-S. Li, X.-J. Sang, W.-L. Chen, L.-C. Zhang, Z.-M. Zhu, T.-Y. Ma, Z.-M. Su, E.-B. Wang, Enhanced Visible Photovoltaic Response of TiO_2 Thin Film with an All-Inorganic Donor–Acceptor Type Polyoxometalate. *ACS Appl. Mater. Interfaces* **2015**, *7*, 13714–13721.

[31] X. Lopez, J. A. Fernandez, J. M. Poblet. Redox Properties of Polyoxometalates: New Insights on the Anion Charge Effect. *Dalton. Trans.* **2006**, *9*, 1162-1167.

[32] Y. C. Li, H. Z. Zhong, R. Li, Y. Zhou, C. H. Yang, Y. C. Li, High-Yield Fabrication and Electrochemical Characterization of Tetrapodal CdSe, CdTe, and $CdSe_xTe_{1-x}$ Nanocrystals. *Adv. Funct. Mater*. **2006**, *16*, 1705-1716.

[33] J. W. Vickers, H. Lv, J. M. Sumliner, G. Zhu, Z. Luo, D. G. Musaev, Y. V. Geletii, C. L. Hill, Differentiating Homogeneous and Heterogeneous Water Oxidation Catalysis: Confirmation that $[Co_4(H_2O)_2(\alpha\text{-}PW_9O_{34})_2]^{10-}$ Is a Molecular Water Oxidation Catalyst. *J. Am. Chem. Soc*. **2013**, *135*, 14110-14118.
